# Towards Artificial Intelligence Augmenting Facilitation: AI Affordances in Macro-Task Crowdsourcing

**DOI:** 10.1007/s10726-022-09801-1

**Published:** 2023-01-10

**Authors:** Henner Gimpel, Vanessa Graf-Seyfried, Robert Laubacher, Oliver Meindl

**Affiliations:** 1 Research Center Finance & Information Management, University of Hohenheim - Digital Management, Fraunhofer FIT - Branch Business & Information Systems Engineering, Stuttgart, Germany; 2 Research Center Finance & Information Management, Branch Business & Information Systems Engineering of the Fraunhofer FIT, Augsburg, Germany; 3 Center for Collective Intelligence, Massachusetts Institute of Technology (MIT), Cambridge, MA United States of America

**Keywords:** Affordance, Artificial Intelligence, Facilitation, Macro-Task Crowdsourcing

## Abstract

Crowdsourcing holds great potential: macro-task crowdsourcing can, for example, contribute to work addressing climate change. Macro-task crowdsourcing aims to use the wisdom of a crowd to tackle non-trivial tasks such as wicked problems. However, macro-task crowdsourcing is labor-intensive and complex to facilitate, which limits its efficiency, effectiveness, and use. Technological advancements in artificial intelligence (AI) might overcome these limits by supporting the facilitation of crowdsourcing. However, AI’s potential for macro-task crowdsourcing facilitation needs to be better understood for this to happen. Here, we turn to affordance theory to develop this understanding. Affordances help us describe action possibilities that characterize the relationship between the facilitator and AI, within macro-task crowdsourcing. We follow a two-stage, bottom-up approach: The initial development stage is based on a structured analysis of academic literature. The subsequent validation & refinement stage includes two observed macro-task crowdsourcing initiatives and six expert interviews. From our analysis, we derive seven AI affordances that support 17 facilitation activities in macro-task crowdsourcing. We also identify specific manifestations that illustrate the affordances. Our findings increase the scholarly understanding of macro-task crowdsourcing and advance the discourse on facilitation. Further, they help practitioners identify potential ways to integrate AI into crowdsourcing facilitation. These results could improve the efficiency of facilitation activities and the effectiveness of macro-task crowdsourcing.

## Introduction

A﻿rtificial intelligence (AI) holds the potential to transform collaborative activities such as crowdsourcing (Griffith et al. 2019; Introne et al. 2011; Kiruthika et al. 2020; Manyika et al. 2016; Seeber et al. 2020). In crowdsourcing, a crowd collaborates to solve a task in a digital participative environment, such as an online platform (Estellés-Arolas and González-Ladrón-de-Guevara 2012). Thereby, the crowd may be diverse, including individuals from diverse disciplinary backgrounds (Cullina et al. 2015; Dissanayake et al. 2019). When a crowd is dedicated to tackling complex and interdependent tasks collaboratively, the practice is referred to as macro-task crowdsourcing (Robert 2019; Schmitz and Lykourentzou 2018). Macro-tasks are tasks that are difficult or sometimes impossible to decompose into smaller (interdependent) subtasks (Robert 2019). The use of crowdsourcing to address macro-tasks is rarely straightforward and requires a specific skill set and knowledge of the crowd (Schmitz and Lykourentzou 2018). A prominent example of macro-tasks are wicked problems. Wicked problems are highly complex and thus require the involvement of many different stakeholders (Alford and Head 2017; Head and Alford 2015; Ooms and Piepenbrink 2021). Global challenges that are very broad in scope, such as the advancement of the sustainable development goals as defined by the United Nations (2015), may be understood as wicked problems of current relevance (McGahan et al. 2021). In response to these problems, existing macro-task crowdsourcing initiatives such as OpenIDEO or Futures CoLab elaborate on sustainability-related improvements and solution approaches (Gimpel et al. 2020; Kohler and Chesbrough 2019).

For macro-task crowdsourcing to realize its potential and tackle such complex problems, structure, guidance, and support are needed to coordinate the collaborating crowd workers (Adla et al. 2011; Azadegan and Kolfschoten 2014; Shafiei Gol et al. 2019). If this need is satisfied through unbiased (human) observation and intervention, it is known as facilitation (Adla et al. 2011; Bostrom et al. 1993). Although facilitation has already been widely analyzed in other contexts such as group interaction (Bostrom et al. 1993), face-to-face meetings (Azadegan and Kolfschoten 2014), and open innovation (Winkler et al. 2020), it has barely been investigated in macro-task crowdsourcing. AI is seen as a system’s ability to interpret and learn from external data to achieve a predetermined goal (Kaplan and Haenlein 2019). With AI breaking human text challenges (Wang et al. 2019), new potentials arise, especially for text-based applications like crowdsourcing. The high transformative potential of AI gives rise to the question: Can AI support the facilitation of macro-task crowdsourcing? If it can, the quality of crowdsourcing results might be improved, leading to better outcomes and results. For example, an AI with semantic text understanding could recognize novel or innovative-yet-unrecognized ideas and highlight these as focal points for further discussion within the crowd (Toubia and Netzer 2017). Furthermore, by relieving the bottleneck of labor- and knowledge-intensive facilitation, macro-task crowdsourcing could be applied to more wicked problems.

AI and facilitation may be closely interwoven in macro-task crowdsourcing. Facilitation, in the specific context of macro-task crowdsourcing, requires human facilitators as well as technological advancements which support the former by fulfilling a large variety of burdensome activities (Briggs et al. 2013; de Vreede and Briggs 2019; Franco and Nielsen 2018; Khalifa et al. 2002; Seeber et al. 2016; Winkler et al. 2020). Among other duties, a facilitator is responsible for understanding the problem to be tackled by the macro-task crowdsourcing, motivating and guiding the crowd and its dialogues, and making sense of the outcome. Lately, AI – as one specific technological advancement – has been investigated for its supportive potential (Rhyn and Blohm 2017; Seeber et al. 2016; Tavanapour and Bittner 2018a). AI carries various functionalities, including text mining or natural language processing, that can support macro-task crowdsourcing facilitation. For instance, intelligent conversational agent systems could guide the crowd through the crowdsourcing process (Derrick et al. 2013; Ito et al. 2021) or issue detailed instructions to crowd workers in the form of specific tasks (Qiao et al. 2018). The evaluation of the workers’ contributions could also be drastically simplified by designing appropriate systems that leverage the potential of text mining and natural language generation to automatically generate reports or summaries (Füller et al. 2021; Rhyn et al. 2020). Such AI-augmented facilitation systems could improve human facilitation (Adla et al. 2011; Siemon 2022), (partially) automate facilitation processes (Gimpel et al. 2020; Jalowski et al. 2019; Kolfschoten et al. 2011), or even wholly replace the facilitator with an AI agent (de Vreede and Briggs 2019).

Although AI has considerable potential in macro-task crowdsourcing and assisting human problem solving (Rhyn and Blohm 2017; Schoormann et al. 2021; Seeber et al. 2020), there are only a few AI-related contributions in the literature on macro-task crowdsourcing or crowdsourcing facilitation. A holistic understanding of how AI could be applied to facilitate problem-solving in on- or offline groups is missing. However, such a holistic understanding is necessary to guide further research on crowdsourcing and facilitation and inform practitioners as to how crowdsourcing initiatives might be improved. We set out to investigate how AI can and may enable macro-task crowdsourcing facilitation. Therefore, we pose the following research questions (RQs), which address both the identified lack of research into macro-task crowdsourcing facilitation and the need for a holistic understanding of AI in this given context:

*RQ1: Which activities comprise macro-task crowdsourcing facilitation?*

*RQ2: What action possibilities does AI afford for macro-task crowdsourcing facilitation?*

We apply a two-stage, bottom-up approach to establish a theory-driven understanding validated and refined using practical insight to answer these research questions. In our approach, we turn to affordance theory (Volkoff and Strong 2017), which is known to help develop better theories in IT-associated transformational contexts (Ostern and Rosemann 2021). Given AI’s high potential to transform crowdsourcing, affordance theory can be seen as an established, suitable, and meaningful lens to theorize the relationship between the technological artifact of AI and the goal-oriented actor – namely, the facilitator (Lehrer et al. 2018; Markus and Silver 2008; Ostern and Rosemann 2021; Volkoff and Strong 2013). In the first stage, we develop initial sets of macro-task crowdsourcing facilitation activities and AI affordances. Both sets are based on a structured search and review of extant scholarly knowledge. The second stage validates and refines our facilitation activities and AI affordances. We observe two real-world macro-task crowdsourcing initiatives and perform six interviews with experts from the crowdsourcing facilitation and AI domain, thus, including insights from practice.

For RQ1, our results provide a detailed understanding of macro-task crowdsourcing facilitation comprising 17 facilitation activities. We answer RQ2 by developing a set of seven AI affordances relevant to macro-task crowdsourcing facilitation. We also detail manifestations of the affordances that demonstrate actionable practices of AI-augmented macro-task crowdsourcing facilitation. Our findings increase the scholarly understanding of macro-task crowdsourcing facilitation and the application of AI therein. Furthermore, the results will help practitioners to evaluate potential ways of integrating AI in crowdsourcing facilitation. These results will increase the efficiency of facilitation activities and, ultimately, increase the effectiveness of macro-task crowdsourcing.

The remainder of the paper is structured as follows: Sect. 2 provides theoretical background on macro-task crowdsourcing facilitation, AI-augmented facilitation, and affordance theory. We outline our research process in Sect. 3. Section 4 presents the macro-task crowdsourcing facilitation activities, the AI affordances, and the manifestations of AI in macro-task crowdsourcing facilitation. After discussing the implications and limitations of our results in Sect. 5, we conclude with a brief summary in Sect. 6.

## Theoretical Background

### Macro-Task Crowdsourcing Facilitation

#### Macro-Task Crowdsourcing

Crowdsourcing is an umbrella term that can have many meanings. The concept was first introduced in an article in Wired magazine (Howe 2006b). Elsewhere, Howe (2006a) defines crowdsourcing as “the act of a company or institution taking a function once performed by employees and outsourcing it to an undefined (and generally large) network of people in the form of an open call.” Since then, understandings of crowdsourcing have evolved. Estellés-Arolas and González-Ladrón-de-Guevara (2012) proposed a holistic definition that we will use in this paper:

*“Crowdsourcing is a type of participative online activity in which an individual, an institution, a non-profit organization, or company proposes to a group of individuals of varying knowledge, heterogeneity, and number, via a flexible open call, the voluntary undertaking of a task.”*

A panoply of different crowdsourcing types exists, ranging from corporate to social or public contexts (Vianna et al. 2019). In a corporate context, open innovation is used to strategically manage knowledge flows between an external crowd and a firm to improve the firm’s innovation processes (Bogers et al. 2018). With crowdfunding, entrepreneurs can receive funding, via an open call, from funders who may receive a private benefit in return (Belleflamme et al. 2014). Organizations can use micro-task crowdsourcing to outsource low-complexity tasks (e.g., image tagging, or phone number verification) completed by independent crowd workers (Hossain and Kauranen 2015; Schenk and Guittard 2011). More complex tasks (e.g., invention or software engineering) require collaboration among crowd workers (Kittur et al. 2013). Flash organizations, for example, are computationally built structures comprised of a crowd automatically arranged into a hierarchy, where participants are assigned to smaller units focused on complex tasks according to their particular skills (Valentine et al. 2017). The structure of the resultant crowd organization can adapt over time, allowing it to efficiently collaborate and achieve open-ended goals relating to complex tasks (Retelny et al. 2014; Valentine et al. 2017). Real-world problems can be approached by using citizen science, a participative way of performing research involving experts and non-experts (Hossain and Kauranen 2015; Wiggins and Crowston 2011). For example, Fritz et al. (2019) underline the scientific value of citizens’ contributions of data which helped to track the progress of the United Nations’ sustainable development goals.

As a step beyond predominant crowdsourcing types, we define *macro-task crowdsourcing* based on Leimeister (2010), Lykourentzou et al. (2019), Malone et al. (2010), and Vianna et al. (2019):

*Macro-task crowdsourcing leverages the collective intelligence of a crowd through facilitated collaboration on a digital platform to address complex or wicked problems.*

The problems being addressed with the help of macro-task crowdsourcing may range from open innovation product design, to software development, or to grand social challenges (Kohler and Chesbrough 2019; McGahan et al. 2021) like climate change (Introne et al. 2011). Many of these are rooted in wicked problems characterized by their high complexity and their need to elicit broad stakeholder involvement (Alford and Head 2017; Ooms and Piepenbrink 2021). Macro-task crowdsourcing differs from existing crowdsourcing types in several ways. Although the boundaries between macro-task and, for example, micro-task crowdsourcing are blurred, there are some distinguishing characteristics, which are presented in Table 1.

**Table 1:** Distinctions Between Micro- and Macro-Task Crowdsourcing

Dimension	Micro-Task Crowdsourcing	Macro-Task Crowdsourcing
Nature of Problem	Well-defined, structured, and decomposable into smaller parts, which requires low stakeholder involvement	Ill-defined with no clear structure and low decomposability, which requires broad stakeholder involvement
Contribution Creation	Parallelized collection of contributions with a low level of collaboration	Collaborative and iterative exchange of ideas among (groups) of workers
Crowd Requirements	Workers with skills aligned explicitly to the problem and high efficiency in task-completion	Workers with different backgrounds, diversity in their domain expertise, and a high willingness to collaborate
Guiding Process	The requestor or the digital platform’s algorithm performs repetitive and standardized patterns of actions	The facilitator or facilitating teams guide process phases with high degrees of freedom for the workers
Generated Outcome	Aggregable and structurable solutions to the problem	Approaches to addressing the problem, which are difficult to synthesize

The fact that the problem cannot easily be broken down into smaller constituent parts means it requires a high level of crowd diversity – i.e., providing multiple perspectives from experts with different levels of expertise and knowledge in various disciplines (Lykourentzou et al. 2019; Robert 2019). Due to the complexity of the underlying problem and the broad stakeholder involvement, a guiding, moderating, and neutral central agent is necessary, which we refer to as a facilitator (Gimpel et al. 2020). It is important to note that the results produced by the crowd will not necessarily be the final solution to the overarching problem. Existing macro-task crowdsourcing initiatives such as Climate CoLab (Introne et al. 2013), Futures CoLab (Gimpel et al. 2020), and OpenIDEO (Kohler and Chesbrough 2019) tend to produce valuable but non-conclusive approaches to addressing a wicked problem from one specific angle. These approaches have evolved and matured during several guided phases (Gimpel et al. 2020; Introne et al. 2013), making macro-task crowdsourcing even more reliant on a facilitator and a clear understanding of its role within the crowdsourcing initiative.

**Fig. 1:**
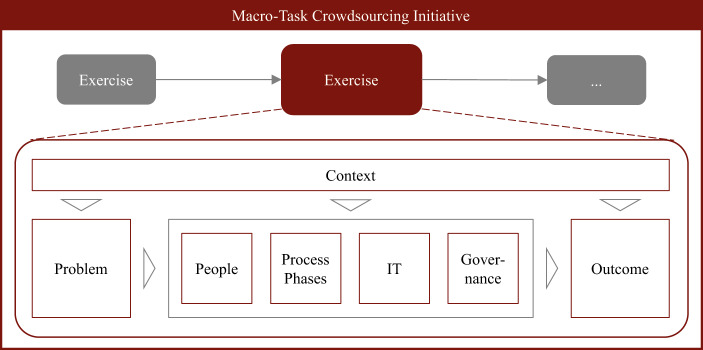
Terminology Within a Macro-Task Crowdsourcing Initiative

The panoply of different crowdsourcing types has produced a variety of terminologies with synonyms and ambiguities now requiring unification. Figure 1 depicts an abstract view of existing terms and definitions within crowdsourcing. Generally, we use the term macro-task crowdsourcing *initiative* to refer to an overarching set of online activities that aim to address a problem (Estellés-Arolas and González-Ladrón-de-Guevara 2012). We refer to a crowdsourcing *exercise* as a whole process of crowdsourcing techniques (Vukovic and Bartolini 2010) that may be applied multiple times or in combination with other exercises as part of a macro-task crowdsourcing initiative.

The *context* of an exercise is highly relevant. A macro-task crowdsourcing initiative may conduct multiple exercises in various (e.g., geographical) environments, using different strategies or workflows (e.g., to address the problem) with different infrastructural prerequisites (e.g., hard- and software). The nature of the *problem* being tackled by an exercise influences how a task is designed and, therefore, how contributions are generated (Zuchowski et al. 2016).

In an exercise, three different groups of *people* participate. The requestor is an organization or an individual that seeks help, the worker is part of a help-offering crowd capable of (partially) addressing or solving the requestor’s problem (Pedersen et al. 2013). The facilitator acts as a crucial intermediary who tries to understand the requestor and facilitates the crowd of workers to reach a predefined goal concerning the problem (Franco and Nielsen 2018; Gimpel et al. 2020; Rippa et al. 2016). From an activity-driven perspective, exercises consist of three major *process phases*: preparation, execution, and resolution. While preparation refers to “breaking down a problem or a goal into lower level, smaller sub-task” (Vukovic and Bartolini 2010), execution describes the elaboration on the task by a diverse crowd of workers (Zuchowski et al. 2016) supported and guided by one or more facilitators. Evaluating and synthesizing the workers’ contributions finishes the last process phase, termed resolution (Lopez et al. 2010). *IT* enables all participants to collaborate online in a distributed or decentralized way. Typically, a digital platform is used to capture and store the interactions and communication between individuals (Lopez et al. 2010). Interactions on a digital platform for macro-task crowdsourcing can include rating, creation, solving, and processing (Geiger and Schader 2014). Sometimes tools like video communication software are used to run the exercise more efficiently or effectively. To steer the exercise within the given context, *governance*, using a dedicated strategy, creates suitable boundary conditions for the people (Blohm et al. 2018; Pedersen et al. 2013). While rules can define norms or desired conducts, roles govern responsibilities and accountabilities, and culture creates a desirable and productive atmosphere for collaboration.

Each exercise results in different types of *outcomes* (Zuchowski et al. 2016). Contributions represent manifestations of work on the communicated and processed task. We see tacit knowledge gained during the exercises as learnings that could, for instance, be achieved by reflections or feedback. We distinguish these from consequences, which represent immutable conclusions that have been caused by performing the exercise (e.g., unsatisfied workers who will not contribute to future exercises). Finally, every stakeholder of the macro-task crowdsourcing initiative can perceive value in the exercise. While the requestor could, for example, see value in the synthesized contributions, a worker could perceive value in social recognition within the crowd.

Behind each of these terms, there is a whole range of activities that, taken together, should be carefully aligned with a goal during the macro-task crowdsourcing initiative, in order to contribute to the overarching problem. Thus, facilitation is important to ensure proper alignment and goal orientation. Thereby, facilitators play an essential role, particularly – yet, not only – during the exercises.

#### Facilitation in Crowdsourcing

In the crowdsourcing domain, crowdsourcing governance aims to facilitate workers in performing their tasks and steer them toward a solution (Pedersen et al. 2013; Shafiei Gol et al. 2019). According to Shafiei Gol et al. (2019), whether crowdsourcing governance is centralized or decentralized, the task is to control and coordinate workers on the crowdsourcing platform. This involves activities such as defining the task (Zogaj and Bretschneider 2014), providing proper incentives (Vukovic et al. 2010), ensuring the quality of the contributions (Blohm et al. 2018), and managing the community and its culture (Zuchowski et al. 2016). Crowdsourcing governance is often analyzed in environments involving paid work or smaller tasks (Blohm et al. 2018; Shafiei Gol et al. 2019). Hence, activities like controlling costs and standardizing procedures also gain relevance (Shafiei Gol et al. 2019). Despite extensive frameworks (Blohm et al. 2020; Shafiei Gol et al. 2019; Zogaj et al. 2015), crowdsourcing governance is often conceptualized on the organizational and platform level, which could explain why it is also referred to as a management activity (Blohm et al. 2018; Jespersen 2018; Pohlisch 2021; Zogaj and Bretschneider 2014). The increasing complexity of the problems under investigation means increasingly sophisticated governance strategies are required to deliver successful crowdsourcing initiatives (Blohm et al. 2018; Boughzala et al. 2014; Pedersen et al. 2013). Since macro-task crowdsourcing initiatives are known for their complex (sometimes even wicked) underlying problems in a collaborative environment, utilizing facilitation can be a suitable and effective governance strategy. Facilitation is primarily focused on the crowd, enabling workers to collaborate on complex tasks and, ultimately, reach an overarching goal (Gimpel et al. 2020; Kim and Robert 2019; Lykourentzou et al. 2019).

To tackle increasingly complex – often wicked – problems using macro-task crowdsourcing, the facilitation of groups is both highly relevant and very challenging (Khalifa et al. 2002; Shafiei Gol et al. 2019). Following Bostrom et al. (1993), the main aim of facilitation is to ensure unified goal orientation among collaborating workers. This challenging task can require various social and technical skills or abilities to support problem-solving (Antunes and Ho 2001). Researchers have explored several types of facilitation specifically tailored to collaborative settings. Adla et al. (2011) differentiate between four overlapping types: Technical facilitation mainly aims to support participants with technology issues. Group process facilitation strives to ensure all members of a group jointly reach overarching goals such as motivation or moderation. Process facilitation assists by coordinating participants or structuring meetings. Finally, content facilitation focuses on, and introduces changes to, the content under discussion. Facilitators serve as experts practicing techniques to support problem-solving processes (Winkler et al. 2020), for example, in face-to-face meetings (Azadegan and Kolfschoten 2014; Bostrom et al. 1993). Besides completing a burdensome amount of work before, during, and after the collaboration (Vivacqua et al. 2011), facilitators must also evince particular character and behavioral traits (Dissanayake et al. 2015a). Training and experience (Clawson and Bostrom 1996), appearance and behavior within a group (Franco and Nielsen 2018; Ito et al. 2021; McCardle-Keurentjes and Rouwette 2018), and the handling of feedback and reflection (Azadegan and Kolfschoten 2014; de Vreede et al. 2002) play an essential role here. Thereby, facilitators maintain a delicate balance between situations in which they moderate and observe the group and instances in which they intervene – for instance, due to content-related issues (Khalifa et al. 2002) – without compromising the outcome of the exercise (Dissanayake et al. 2015b). To better assist the group and balance the workload, multiple facilitators with different foci may sometimes be involved, making it possible to split the work among the facilitators (Franco and Nielsen 2018) and maintain a good relationship with all the workers (Liu et al. 2016). However, some scholars note that face-to-face facilitation techniques may be less effective when applied in distributed or virtual environments (Adla et al. 2011). Hence, it is difficult for crowdsourcing facilitators to rely on facilitation knowledge established in other contexts. This difficulty could be rooted in the fundamentally different nature of collaboration on a crowdsourcing platform (Gimpel et al. 2020; Nguyen et al. 2013).

Building upon the current, broad understanding of crowdsourcing governance (Blohm et al. 2020; Pedersen et al. 2013; Shafiei Gol et al. 2019) and facilitation (Antunes and Ho 2001; Bruno et al. 2003; Kolfschoten et al. 2011; Maister and Lovelock 1982; Zajonc 1965) offered in the literature – in particular, an existing definition by Bostrom et al. (1993) – we define macro-task crowdsourcing facilitation thus:

*Facilitation in macro-task crowdsourcing initiatives comprises all observing and intervening activities used before, during, and after a macro-task crowdsourcing exercise to foster beneficial interactions among crowd workers aimed at making (interim-) outcomes easier to achieve and ultimately align joint actions with predefined goals.*

Despite substantial knowledge of crowdsourcing governance and facilitation, an overarching and integrated understanding of relevant facilitation activities in macro-task crowdsourcing is missing. Therefore, it is challenging for facilitators to delimit their competencies in crowdsourcing endeavors involving many participants and perspectives (Zhao and Zhu 2014).

### Advances in AI-Augmented Facilitation

AI uses technologies and algorithms to simulate and replicate human behavior or achieve intelligent capabilities (Alsheibani et al. 2018; Simon 1995; Stone et al. 2016; Te’eni et al. 2019). AI may be defined as a “[…] system’s ability to correctly interpret external data, to learn from such data, and to use those learnings to achieve specific goals and tasks through flexible adaptation […]” (Kaplan and Haenlein 2019). Although more general definitions exist – such as those by Rai et al. (2019) and Russell and Norvig (2021) – in this paper, we follow the definition by Kaplan and Haenlein (2019). Their socio-technical system perspective focuses on the interrelationship between humans and AI, which is highly relevant in the context of macro-task crowdsourcing facilitation. While AI has been a subject established in science for over seven decades (Haenlein and Kaplan 2019; Rzepka and Berger 2018; Simon 1995), in recent years, it has received increasing attention in both research and practice (Bawack et al. 2019; de Vreede et al. 2020; Hinsen et al. 2022; Hofmann et al. 2021; Leal Filho et al. 2022; Pumplun et al. 2019; Rai 2020). AI is expected to disrupt the interplay between user, task, and technology (Maedche et al. 2019; Rzepka and Berger 2018) and the nature of work (Brynjolfsson et al. 2017; Iansiti and Lakhani 2020; Nascimento et al. 2018). This expectation is accompanied by many unrealistic expectations, and the timeless question of “[W]hat can AI do today?” (Brynjolfsson and McAffe 2017). There is a stream of AI research that answers this question using terminology usually related to humans or animals, including intelligence, learning, recognizing, and comprehending (Asatiani et al. 2021; Benbya et al. 2021; Rai et al. 2019), that explicitly considers human-inspired AI and humanized AI (Kaplan and Haenlein 2019). For example, Hofmann et al. (2020) answer the question of “what can AI do today?” by providing a structured method to create AI use-cases applicable to various domains. Thereby, they distinguish seven abstract functions, defined in Table 2, through which AI can occur as a solution: perceiving, identification, reasoning, predicting, decision-making, generating, and acting (Hofmann et al. 2020). However, such approaches may also lead to the over-humanization of AI and should not distort the fact that AI systems are human-made artifacts, not humans.

**Table 2:** Seven Artificial Intelligence Functions, Following Hofmann et al. (2020)

AI Function	Definition
Perceiving	“Acquiring and processing data from the real world to produce information”
Identification	“Extracting and identifying specific objects from data”
Reasoning	“Explaining underlying relationships and structures in data”
Prediction	“Estimating future events or conditions on a continuous scale”
Decision-making	“Choosing between known, discrete alternatives”
Generating	“Producing or creating something”
Acting	“Executing goal-oriented actions (e.g., movement, navigate, control)”

AI differs in the presence of cognitive, emotional, or social intelligence (Kaplan and Haenlein 2019). To be able to best support – or even replace – the facilitator would require an AI to hold all three types of intelligence, thereby resulting in a self-conscious and self-aware humanized AI (de Vreede and Briggs 2019; Kaplan and Haenlein 2019). Humanized AIs are not yet available in the facilitation domain, which could either be due to the complexity of collaboration (Kolfschoten et al. 2007) or the limited capabilities of current AI systems (Briggs et al. 2013; Kaplan and Haenlein 2019; Sousa and Rocha 2020). Hence, scholars from the facilitation domain focus on projects and approaches to building human-inspired AIs (Seeber et al. 2018). We refer to these as AI-augmented facilitation systems, which could have a vast impact on team collaboration (Maedche et al. 2019; Seeber et al. 2018, 2020). For instance, Derrick et al. (2013) and Ito et al. (2021) propose the first results of conversational AI capable of issuing instructions to team members or responding to workers’ contributions. Further inspired by the widespread application of AI (Dwivedi et al. 2021; Wilson and Daugherty 2018), researchers have also begun to explore more specifically AI’s potential use in crowdsourcing facilitation (de Vreede and Briggs 2018; Rhyn and Blohm 2017; Tavanapour and Bittner 2018a). For instance, some approaches seek to automate facilitation activities and decision-making by integrating AI such as text mining or natural language processing (Gimpel et al. 2020). Most of these AI-augmented approaches are prototypes, suggesting that further investigation of possible AI-augmented facilitation may be warranted (Askay 2017; Ghezzi et al. 2018; Robert 2019).

### Affordance Theory

Within our research, we use affordance theory as a conceptual lens. Affordances are action possibilities that characterize the relationship between a goal-oriented actor and an artifact within a given environment (Burlamaqui and Dong 2015; Gibson 1977; Markus and Silver 2008). The concept of affordances was initially introduced in ecological psychology to describe how animals perceive value and meanings in things within their environment (Gibson 1977). Scholars have translated the concept of affordances to technological contexts (Achmat and Brown 2019; Autio et al. 2018; Bayer et al. 2020; Gaver 1991). Affordances theory now serves as an established lens to investigate socio-technical phenomena emerging from information technology (Dremel et al. 2020; Du et al. 2019; Keller et al. 2019; Lehrer et al. 2018; Malhotra et al. 2021; Markus and Silver 2008). Thereby, affordances describe the relationship between an actor and an information technology to determine goal-oriented action possibilities available to the actor and using specific information technology at hand (Faik et al. 2020; Markus and Silver 2008; Volkoff and Strong 2017). Actors can perceive or actualize affordances (Ostern and Rosemann 2021). Perceiving affordances requires that the actor holds a certain level of awareness regarding the information technology and is, hence, able to identify its potential uses (Burlamaqui and Dong 2015; Volkoff and Strong 2017). The information about a perceived affordance can lead actors to an affordance’s actualization. Herein, the actor makes efforts to realize the affordance, unleashing the value it holds in relation to the actor’s goal (Ostern and Rosemann 2021).

To analyze the “cues of potential uses” (Burlamaqui and Dong 2015) of AI in a specific environment, researchers often turn to affordance theory (Burlamaqui and Dong 2015; Kampf 2019; Volkoff and Strong 2017). In our endeavor, the particular environment is macro-task crowdsourcing with the facilitator as the actor and AI as the specific information technology. This confluence of technology and actor in our macro-task crowdsourcing context is a complex socio-technical phenomenon, where affordance theory can help better understand the interrelationships. With RQ2, we aim to exploratively investigate the relationship between the actor and the technology, revealing the action possibilities of AI in macro-task crowdsourcing facilitation. In line with the original definition by Gibson (1977) and following technology-related affordance literature (Faik et al. 2020; Leonardi 2011; Norman 1999; Steffen et al. 2019; Vyas et al. 2006), we focus on perceived affordances throughout our research endeavor. Hence, we define affordances in our context as perceived action possibilities arising from AI in macro-task crowdsourcing facilitation that do not necessarily need to be performed (Askay 2017). We see these perceived affordances as necessary to compose the nucleus of AI’s intersubjective meaning for facilitators (Suthers 2006). The most salient perceived affordances will ultimately support collaboration among the crowdsourcing workers.

## Research Design

**Fig. 2:**
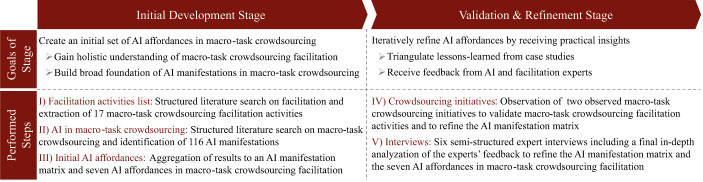
Overarching Two-Stage, Bottom-Up Approach

Our research set out to address the lack of knowledge on macro-task crowdsourcing facilitation and the need for a holistic understanding of how AI might augment facilitation in this context. Thereby, we followed a two-stage, bottom-up approach to establish a theory-driven understanding that we then validated and refined from a practical perspective. In our approach, we turned to affordance theory as an established lens to theorize the relationship between the technological artifact, AI, and the goal-oriented actor, the facilitator (Lehrer et al. 2018; Markus and Silver 2008; Ostern and Rosemann 2021; Volkoff and Strong 2013). Our approach served to identify macro-task crowdsourcing facilitation activities and AI affordances in macro-task crowdsourcing facilitation. Firstly, in the *initial development stage*, we conducted two literature searches. We identified 17 macro-task crowdsourcing activities and 116 statements about AI in macro-task crowdsourcing being further processed to manifestation (i.e., specific action possibilities) that substantiate AI’s potential use for macro-task crowdsourcing facilitation. From this, we identified seven AI affordances for macro-task crowdsourcing. Secondly, we iteratively refined our results in the *validation & refinement* stage through two observed macro-task crowdsourcing initiatives and six semi-structured interviews with experts from the AI and crowdsourcing facilitation domain. Figure 2 depicts the overarching research design, which yielded seven AI affordances for macro-task crowdsourcing.

### Initial Development Stage

We developed an initial set of AI affordances in three steps. The aim in the first two steps was to gain an understanding of facilitation and AI within macro-task crowdsourcing. Thereby, we developed macro-task crowdsourcing facilitation activities necessary for performing the third step, which served to combine relevant insights from literature into an initial set of AI affordances.

In step *I) Facilitation activities list*, we conducted a structured literature search to extract activities that describe macro-task crowdsourcing facilitation. In an initial broad search, we identified the journal ‘Group Decision and Negotiation’ as an adequate source of broad, foundational knowledge about facilitation (Laengle et al. 2018). A searched for the term *‘facilitation’* in this journal returned a total of 176 papers, which we sequentially screened by title, abstract, and full text to determine whether facilitation was the core subject of each article. In doing so, we identified ten papers, plus one additional relevant paper from another outlet (Appendix A.1), whose full-text we further processed. We extracted 477 statements (i.e., excerpts) about activities or capabilities (i.e., repeatable patterns of action) relevant for facilitation. For each statement, we then decided whether the activity or capability was transferable to macro-task crowdsourcing facilitation. We excluded statements if the underlying activity did not necessarily need to be performed by a facilitator (e.g., recruitment of the worker) or if it neither contributed to fostering beneficial interactions among crowd workers or aligning joint actions with predefined goals (e.g., communication of the exercises’ results or distributing rewards to workers). We categorized the 317 remaining statements into 17 broader macro-task crowdsourcing facilitation activities that iteratively emerged in the researcher team’s discussions. These 17 activities served as comprehensive, foundational knowledge about macro-task crowdsourcing facilitation in the next two steps.

Step *II) AI in macro-task crowdsourcing* served to capture manifestations of AI in macro-task crowdsourcing. As highlighted above, the digital nature of crowdsourcing platforms means the application of AI in crowdsourcing is more widespread than in other situations where facilitation plays an essential role (e.g., face-to-face meetings). We conducted a systematic literature review on the topic of macro-task crowdsourcing (vom Brocke et al. 2015; Wolfswinkel et al. 2013). In keeping with our research goal of exploring “cues of potential uses” (Burlamaqui and Dong 2015, p. 305) of AI (i.e., affordances), we had identified ‘information systems,’ ‘computer science,’ and ‘social science’ as our fields of research. Hence, we selected four established databases (i.e., AIS eLibrary, ACM Digital Library, IEEE Explore Digital Library, and Web of Science) that covered this broad disciplinary spectrum. Our search query did not include specific AI terms since the literature includes various definitions and terms to refer to corresponding AI technologies (Bawack et al. 2019). Instead, we iteratively developed our search query and ended up with a more general tripartite version representing a process-driven perspective on online crowdsourcing:

(‘crowd*’ OR ‘collective intelligence’) AND (‘task’ OR ‘activity’ OR ‘action” OR ‘process’ OR ‘capability’ OR ‘facilitat*’) AND (‘platform’ OR ‘information system’ OR ‘information technology’ OR ‘information and communications technology’).

Applying this search query to the identified databases resulted in a total of 5,808 hits. To refine our sample of papers, we identified and removed 502 duplicates, which led to 5,306 distinct papers. In manually screening the papers, we applied the criteria listed in Table 3 to narrow our search results to macro-task crowdsourcing and ensure high levels of relevance and rigor.

**Table 3:** In- and Exclusion Criteria of the Literature Search

Inclusion Criteria	Exclusion Criteria
• Explains induced or abstracted knowledge from multiple crowdsourcing exercises • Contains managing actions performed ex-ante, ex-nunc, or ex-post of a crowdsourcing exercise • Depicts human interaction or collaboration on or with the crowdsourcing platform • Includes frameworks, models, taxonomies, or conceptualizations related to the crowdsourcing- domain	• Does not mainly focus on (macro-task) crowd-sourcing • Is not written in English • Was published before 2000 (and, thus, does not discuss contemporary AI systems) • Is a book, (extended) abstract, presentation, single case study, or research-in-progress paper that does not contain relevant interim results or findings • Has identical authors and elaborates on a very similar topic to a paper already included

Using these criteria, we narrowed the search results by sequentially analyzing title and abstract, which narrowed the total to 283 papers potentially relevant to macro-task crowdsourcing. We read these 283 papers in full text, finally identifying nine papers that name and describe AI in the context of macro-task crowdsourcing. We also included three papers, found elsewhere during our research process, that matched all of our defined criteria. We analyzed these 12 papers (Appendix A.2) in-depth to extract 116 statements about AI manifestations in macro-task crowdsourcing. In the next step, these statements were used together with the previous results to develop AI affordances.

In step *III) Initial AI affordances*, we developed an initial set of AI affordances by combining and aggregating the results of steps I) and II). Thereby, we assigned 116 manifestations (Appendix A.3) of AI in macro-task crowdsourcing to the 17 activities of macro-task crowdsourcing facilitation. To further distinguish and explain the role of AI in each manifestation, we used AI functions proposed by Hofmann et al. (2020). In doing so, we assigned each manifestation one specific AI function, describing how AI occurs or could occur as a solution in the selected manifestation (Hofmann et al. 2020). This two-dimensional matrix resulted in an AI manifestation mapping for macro-task crowdsourcing facilitation.

To create the initial set of affordances, the research team held discussions to identify archetypes within the manifestation mapping. We remained open-minded about whether an archetype would be created based on the functioning of AI (horizontal axis of the matrix) or the facilitation actions (vertical axis of the matrix). To support the development of AI affordances, we reached out to three scholars with expertise in affordance theory. They contributed valuable input regarding common pitfalls and best practices during the development stage. We recognized seven archetypes whose manifestations we then analyzed to identify affordances. Every affordance is described and classified in terms of AI functions and facilitation activity (see Sect. 4.1).

### Validation & Refinement Stage

Although we rigorously identified our AI affordances based on scholarly knowledge, a practical validation was necessary to ascertain potential end-users’ perceptions. To this end, we validated and refined our initial set of AI affordances from step III) with two observed macro-task crowdsourcing initiatives as well as six semi-structured interviews (Myers and Newman 2007).

In step *IV) Crowdsourcing initiatives*, we longitudinally observed two macro-task crowdsourcing initiatives, namely Trust CoLab (TCL) and Pandemic Supermind (PSM). Observing these initiatives not only helped us to validate our facilitation activities but also to gain rare practical insights on macro-task crowdsourcing facilitation and real-world AI manifestations. Table 4 describes the two macro-task crowdsourcing initiatives under consideration.

**Table 4:** Two Macro-Task Crowdsourcing Initiatives Within Validation and Refinement Stage

	Trust CoLab	Pandemic Supermind
**Problem/Goal**	Anticipating the state of trust in medicine and healthcare in 2040	Identifying the critical unmet needs of the COVID-19 pandemic
**Participants**	• 105 workers • 1 facilitator and 1 supporting team	• 206 workers • 2 facilitators and 2 supporting teams
**Usage of AI**	• Ex-post decision to use AI • Semantical clustering of submitted contributions	• Ex-ante decision to use AI • In-situ analysis of contributions, worker activity, and worker network • Extensive semantic evaluation of the contributions

Our primary sources of data collection were documentation (e.g., mails, final reports, and meeting protocols) and participant observation (e.g., discussion within the facilitation team and analysis of AI tools used) that we gained from both crowdsourcing initiatives. Observation of the facilitators‘ actions in the macro-task crowdsourcing initiatives supported the set of 17 facilitation activities from step I). Each of the activities was observed, and no other major activities were found. Additionally, we could refine and enhance the manifestations within our AI manifestation mapping, which was created in step III), by analyzing the application of AI tools and the perceived demand for AI support within both initiatives. Nevertheless, the limited application of AI tools in both initiatives could not validate all affordances and suggested an additional validation and refinement step. Hence, in *step V) Interviews*, we conducted six semi-structured interviews, which we used to uncover potential affordances (Volkoff and Strong 2013). We selected experts from academia and practice with multiple years of experience in the AI or facilitation domain, as listed in Table 5 (Myers and Newman 2007; Schultze and Avital 2011).

**Table 5:** Experts for Validation Interviews

ID	Focus	Experience With the Focus	Job Title
1	Intersect Facilitation and Artificial Intelligence	2 years	Researcher
2	Intersect Facilitation and Artificial Intelligence	2 years	Researcher
3	Artificial Intelligence	4 years	AI Developer
4	Artificial Intelligence	7 years	Co-Founder of AI Start-up
5	Facilitation	6 years	Manager
6	Facilitation	5 years	Project Director

Interviews lasted between 37 and 72 min, were held in the native language of the interviewee, and were recorded with the consent of each interviewee. We informed the interviewees about the research topic and sent a detailed interview guide in advance to better allow the interviewees to prepare for the interview. The guide contained definitions and illustrations, the then-current set of affordances, and the intended structure of the interview. Appendix C.1 provides more details about the structure of the interview as well as the prepared questions.

The semi-structured interviews started with a short description of the research project and definitions of crowdsourcing and facilitation necessary to ensure a mutual understanding of crowdsourcing facilitation. After that, we encouraged the interviewees to share their experience of AI within an ideation section (i.e., a less structured and guided part of the interview). Next, we sought open-ended feedback on the affordances by asking questions regarding the completeness, comprehensiveness, meaningfulness, level of detail, and applicability of the criteria in relation to today’s crowdsourcing initiatives (Sonnenberg and vom Brocke 2012). During the interviews, we took notes to highlight the experts’ essential statements and better respond to the interviewee in the course of the conversation. We iteratively adapted and refined our affordances after each interview. The experts’ feedback led us to overhaul one affordance entirely (i.e., workflow enrichment; previously: environment creation) and improve the descriptions of two other affordances (i.e., improvement triggering and worker profiling).

To align all of the practical and theoretical insights gained, we conducted a final reflective refinement after the interviews. Therein, we followed Schreier (2012) to carefully analyze all six experts’ statements regarding our predefined criteria and enrich our AI manifestation, mapping with potential use cases of AI within macro-task crowdsourcing facilitation named by the experts. Appendix C.3 contains some exemplary expert quotes. Our concept-driven coding frame (Schreier 2012) comprised two categories: (1) feedback regarding artificial intelligence affordances and (2) potential use-cases of AI within macro-task crowdsourcing facilitation. While the feedback is structured in five subcategories according to our defined criteria, the potential use-cases encompass 17 subcategories representing the facilitation activities developed in step I). We extracted transcripts of all relevant statements from the interviewees and mapped these to our coding frame. Finally, we refined the AI affordances and AI manifestation mapping accordingly. The two validation and refinement steps yielded a validated and refined list of seven affordances and 44 manifestations. Appendix B contains a detailed description of the refinement and Appendix C.2 a description of the validation.

## Results

### Macro-Task Crowdsourcing Facilitation

Based on our literature search, we identified 17 macro-task crowdsourcing facilitation activities. Table 6 comprises an exhaustive list of activities found in the current literature, from facilitation joining crowdsourcing’s specific conditions. We argue that the distinction between a more straightforward administrative activity (e.g., sending invitation emails to the workers) and a more complex facilitation activity (e.g., writing a motivational text for the workers’ invitation) can depend on each particular exercise. The borders of this distinction can also be fluid. Nevertheless, it is essential to clearly define the facilitator’s role in each exercise to avoid misunderstandings between the facilitator and other stakeholders of the macro-task crowdsourcing initiative (e.g., the platform administrator or the requestor).

**Table 6:** Facilitation Activities in Macro-Task Crowdsourcing

Activity Name	Description	Supporting Literature
Task Design	Decomposition of an overarching problem into small workable pieces that are bundled into tasks to be presented to the workers	Antunes and Ho (2001), Boughzala et al. (2014), Hetmank (2013), Khalifa et al. (2002), Kolfschoten et al. (2007), Pohlisch (2021), Zogaj and Bretschneider (2014), Zogaj et al. (2015)
Task Communication	Preparation and distribution of relevant information and instructions regarding the tasks, presented in a comprehensible and appealing way	Antunes and Ho (2001), Blohm et al. (2020), Kolfschoten et al. (2011), de Vreede et al. (2002), Erickson et al. (2012), Xia et al. (2015), Zuchowski et al. (2016)
Workflow Design & Selection	Composing a sequence of necessary work steps to be executed on the platform to address the designed tasks by (a team of) workers	Assis Neto and Santos (2018), Briggs et al. (2013), Geiger et al. (2011), Hetmank (2013), Khalifa et al. (2002), Kolfschoten et al. (2007)
Worker Motivation	Triggering workers’ intrinsic or extrinsic motivation in order to stimulate a high rate of contributions and a high level of engagement on the platform	Askay (2017), Adla et al. (2011), Azadegan and Kolfschoten (2014), Blohm et al. (2020), Chittilappilly et al. (2016),Vukovic et al. (2010), de Vreede et al. (2002)
Contribution Support	Assisting the workers in the execution of their tasks through explanations, consultation, or training to foster task completion	Adla et al. (2011), Blohm et al. (2018), de Vreede et al. (2002), Franco and Nielsen (2018), Hosseini et al. (2015), Tavanapour and Bittner (2018b)
Performance Monitoring	Using predefined measurements to measure, analyze, and understand workers’ activity and interactions, as well as the quality of contributions	Blohm et al. (2018), Briggs et al. (2013), Gimpel et al. (2020), Kolfschoten et al. (2011), Nguyen et al. (2015), Vivacqua et al. (2011)
Tool Usage & Integration	Introduction and utilization of (technical) tools to ease the execution of tasks and communication and collaboration among the workers	Briggs et al. (2013), de Vreede et al. (2002), Jespersen (2018), Kolfschoten et al. (2007), Rhyn and Blohm (2017), Tazzini et al. (2013)
Crowd Moderation	Observing and guiding the workers’ communication by understanding group dynamics, recognizing systemic misunderstandings, and identifying or resolving conflicts	Adla et al. (2011), Chan et al. (2016), de Vreede et al. (2002), Faullant and Dolfus (2017), Franco and Nielsen (2018),
Crowd Coordination	Organizing and structuring the joint interaction of the workers by scheduling tasks, managing the workload, and adapting the workflow or strategy when necessary	Antunes and Ho (2001), Askay (2017), Azadegan and Kolfschoten (2014), Franco and Nielsen (2018), Hetmank (2013), Pedersen et al. (2013), Wedel and Ulbrich (2021)
Participation Encouragement	Attracting, nudging, or convincing individual workers to improve their participation or engagement in the exercise	Askay (2017), Azadegan and Kolfschoten (2014), Gimpel et al. (2020), McCardle-Keurentjes and Rouwette (2018), Vivacqua et al. (2011)
Contribution Evaluation	Reviewing, assessing, and filtering relevant contributions using a systematic process	de Vreede et al. (2002), Hetmank (2013), Kolfschoten et al. (2011), McCardle-Keurentjes and Rouwette (2018), Pedersen et al. (2013), Pohlisch (2021), Zhao and Zhu (2016)
Contribution Aggregation	Gathering and collecting information from relevant contributions to meaningfully reassemble or summarize insights gained	Adla et al. (2011), Azadegan and Kolfschoten (2014), Chan et al. (2016), Chittilappilly et al. (2016), Franco and Nielsen (2018), Geiger et al. (2011), Vukicevic et al. (2022)
Quality Control	Analysis of redundant, invalid, or irrelevant contributions in order to learn from workers’ unintended behavior from the workers	Adla et al. (2011), Alabduljabbar and Al-Dossari (2016), Boughzala et al. (2014), Gimpel et al. (2020), Kolfschoten et al. (2011), Zogaj and Bretschneider (2014), Zuchowski et al. (2016)
Decision Making	Elaboration, presentation, and decisions on possible alternatives for action based on the achieved outcomes	Adla et al. (2011), Gimpel et al. (2020), Khalifa et al. (2002), McCardle-Keurentjes and Rouwette (2018), Rhyn and Blohm (2017)
Goal Orientation	Aligning all interactions between workers, facilitators, and requestors on a predefined goal to focus on the purpose of the initiative	Antunes and Ho (2001), Boughzala et al. (2014), Briggs et al. (2013), Gimpel et al. (2020), Khalifa et al. (2002), Kohler and Chesbrough (2019), Pedersen et al. (2013)
Culture Development	Establishing a pleasant atmosphere between and among workers, facilitators, and requestors to achieve efficient and effective communication on the platform	Askay (2017), Azadegan and Kolfschoten (2014), Boughzala et al. (2014) Briggs et al. (2013), de Vreede et al. (2002), Kohler and Chesbrough (2019), Pohlisch (2021)
Risk Management	Identification and evaluation of potential deviations from acceptable behavior on the platform; control and monitor relevant behaviors to foster positive and tackle adverse effects	Kamoun et al. (2015), Kolfschoten et al. (2007), Onuchowska and de Vreede (2018), Pedersen et al. (2013), Pohlisch (2021), Vivacqua et al. (2011), Zogaj and Bretschneider (2014)

In the validation & refinement stage, we observed two macro-task crowdsourcing initiatives (i.e., TCL and PSM). By carefully observing the facilitators within TCL and PSM, we were able to identify action patterns that matched the facilitation activities’ descriptions. Thereby, we confirmed the existence of all 17 facilitation activities, although their scope varied within the two initiatives under consideration. Table 7 depicts exemplary actions in TCL and PSM, mainly performed by the facilitator, that matched the elaborated description of the 17 facilitation activities. Some of these facilitation activities were AI-augmented (i.e., the facilitator was supported by an AI tool), making both initiatives valuable subjects for further analysis regarding our AI affordances.

**Table 7:** Macro-Task Facilitation Activities Within the Selected Initiatives

Activity Name	Exemplary Action in TCL	Exemplary Action in PSM
Task Design	Decomposition of the purpose of the initiative into four sequential exercises, each consisting of one task	Decomposition of the purpose of the initiative into three exercises with a total of five tasks
Task Communication	Discussions about and adaptions of the task to be presented between the facilitator and the supporting team	Provision of exemplary contributions to underline the nature of desired contributions
Workflow Design & Selection	Selection of a four-phase workflow enabled by the platform to develop scenarios about how trust in healthcare or medicine could evolve until 2040	Selection and design of a three-phase workflow (partially) supported by the platform to identify approaches for better pandemic resilience
Worker Motivation	Initial motivational mail that welcomes the workers and highlights the value of the workers’ expected contributions to society	User profile on the platform was prefilled with a short biography of the worker to value the workers’ participation
Contribution Support	Video tutorials and FAQs were designed and made available	Quick responses from the facilitators to questions that arose from the workers
Performance Monitoring	Bi-weekly manual report to track the current amount of workers‘ contributions	Automated AI-augmented dashboard to monitor the contribution upload frequency, most used keywords, and topics arising
Tool Usage & Integration	Usage of one generic online crowdsourcing platform that has been customized to suit the scenario development process	Integration of one AI tool to support the facilitation activities during and after each exercise
Crowd Moderation	Active participation by the supporting team in the discussions and contributions from the worker; reports to the facilitator	Hosting of live virtual events to catalyze conversations about the topics within the ongoing task among the workers
Crowd Coordination	Continuous facilitator notes (notification sent to the crowd) regarding the current and future steps	Creation of worker groups based on their professional background to coordinate parallel task execution in the first exercise; a merging of groups in the second exercise to support cross-fertilization of ideas among workers
Participation Encouragement	Sending targeted emails to workers who were not active on the platform	Weekly encouragement of the crowd via email to send feedback, which was regularly reflected and integrated by the facilitators
Contribution Evaluation	Iterative reviewing and selection of the contributions after each exercise; removal of duplicate contributions	Weekly discussions between the facilitator and the requestor about recent contributions from the workers
Contribution Aggregation	Initial semantical clustering of submitted contributions with manual adaptations	Application of different semantical clustering algorithms and manual refinements
Quality Control	Notifying workers about redundant contributions during the exercises	Continuous monitoring of the social network graph of the crowd to avoid topic biases
Decision Making	Creation of one final report in collaboration with the requestor of the initiative	One detailed report about the results was made publicly available and shared with the requestor
Goal Orientation	Raise discussion-stimulating questions to reach a broad range of sentiment	A small adjustment to one communicated task to cover issues of misunderstanding
Culture Development	General rules regarding behavioral and cultural expectations were made available	Reference to the Chatham House Rule to build an appreciative atmosphere
Risk Management	Test run of the crowdsourcing platform with 10 participants	Thorough testing of the AI tool with data from similar initiatives to ensure the functionality

Throughout the macro-task crowdsourcing initiatives of TCL and PSM, dedicated teams used two different AI tools to support the facilitators in their work. In TCL, the facilitator was mainly supported in aggregating the workers’ contributions between the four phases. All of the workers’ contributions were first exported from the platform before a natural language processing Python script preprocessed the contributions (i.e., performing stemming and lemmatization). The script then created a detailed word cloud to provide the facilitator with a broad overview of the main concepts. Finally, the contributions were semantically clustered by the script using the Universal Sentence Encoder algorithm (Cer et al. 2018). We refer to Appendix D.1 for two interim results of the Python script. The results were discussed by the initiative’s stakeholders and manually refined by the facilitator and the supporting team. In PSM, the two facilitators were supported by a web application written in R. The application could directly access the latest contribution data via an application programming interface provided by the crowdsourcing platform. Therefore, the facilitators could use the web application’s algorithms during internal meetings to discuss the latest contribution data. In TCL, the AI tool only used the codified contributions made by the workers while the web application also used metadata such as comments or likes on the contributions. This metadata allowed a broad set of functionalities such as keyword extraction, topic modeling, word co-occurrences, network analysis, and word searches. We refer to Appendix D.2 for two screenshots of the web application.

### Artificial Intelligence Affordances

Given the extensive knowledge base on macro-task crowdsourcing facilitation, we searched for AI manifestations by conducting a second literature search to create an initial AI manifestation mapping. By analyzing the macro-task crowdsourcing initiatives TCL and PSM regarding potential use-cases of AI-augmented facilitation, and gathering statements about potential use-cases for AI in facilitation from the six expert interviews, we were able to refine and extend our initial AI manifestation mapping. Therein, we searched for archetypes of manifestations that could lead to potential affordances. Table 8 lists the final seven affordances of AI for macro-task crowdsourcing facilitation and one example of how AI-augmented facilitation could be implemented in the case of each affordance.

**Table 8:** Artificial Intelligence Affordances in Macro-Task Crowdsourcing Facilitation

ID	Affordance Name	Description	Exemplary AI Augmentation
**1)**	Contribution Assessment	AI affords in-depth analysis of the quality of workers’ contributions to identify valuable ideas and extract relevant input for further processing.	Semantical natural language processing to remove unnecessary information
**2)**	Improvement Triggering	AI affords identification and nudging of non or less-active workers towards higher participation and triggers improvement measures for inadequate contributions.	Nudging during contribution creation based on natural language understanding
**3)**	Operational Assistance	AI affords support for workers through the whole process of contribution development, including the identification of relevant ideas, elaboration of (interim) results, and submission of the final contribution.	AI chat assistants to answer questions during the contribution creation process
**4)**	Workflow Enrichment	AI affords the provision and integration of useful information and knowledge to a predefined workflow, enabling highly productive collaboration among workers.	Natural language understanding to identify mismatches between the facilitator’s proposed task and the workers’ contributions
**5)**	Collaboration Guidance	AI affords collective guidance for workers during their collaboration on the platform in such a way that they will focus on a predefined goal relating to the overarching problem.	Sentiment detection to generate semantic embeddings of the workers’ contributions
**6)**	Worker Profiling	AI affords analysis of the network of workers to track the skills and activity of individuals as well as to monitor the quality of their created contributions.	(Social) Network algorithms to generate activity reports from the crowdsourcing platform data
**7)**	Decision-making Preparation	AI affords aggregate outcomes and synthesizes relevant contributions and, therefore, creates a valuable foundation for decision-makers.	Summary generation algorithms to synthesize the free-text contributions of the workers

In the following, we describe each of the affordances in detail. Thereby, we explain the relationship between the facilitator’s goal and AI within macro-task crowdsourcing. To further elaborate on the affordances, we highlight some AI manifestations found in the literature (step II), our two macro-task crowdsourcing initiatives (step IV), or our interviews (step V). These manifestations provide examples of what AI is perceived to afford within macro-task crowdsourcing facilitation.

*1) Contribution Assessment.* In bringing a macro-task crowdsourcing initiative to fruition, one of the biggest challenges facilitators face is dealing with the number of contributions made by workers (Blohm et al. 2013; Nagar et al. 2016) and “[understanding] all the results from a crowdsourcing exercise in a way that’s empirical and meaningful” (Expert 5). AI affords the analysis of contributions such that the quality can be assessed and valuable ideas or relevant content can be extracted. Facilitators could use AI to analyze the content of a contribution via semantical natural language processing (Gimpel et al. 2020) to determine its novelty or similarity compared to other contributions. (Semi-)automated contribution assessments could decide whether each contribution brings the initiative one step closer to the goal (Haas et al. 2015; Nagar et al. 2016; Rhyn and Blohm 2017). This could involve removing unnecessary information to allow a better assessment by the facilitator further downstream (Expert 4, 6) or to detect outliers by assessing each contribution’s relevance to the topic at hand (Case PSM).

*2) Improvement Triggering.* In crowdsourcing, facilitators often face a 90-9-1 distribution, where only 1% of the workers create nearly all of the contributions (Troll et al. 2017). Since macro-task crowdsourcing heavily relies on active knowledge exchange and idea cross-fertilization between various workers (Gimpel et al. 2020), non- or low-active workers need to be triggered to contribute, thereby stimulating a better thematic discourse (Expert 2, 4). Yet, even if all workers contribute, their contributions may sometimes lack quality; ideas may lack originality or readability. AI affords recognition of individual contributions that are unoriginal or add no value (e.g., due to the existence of similar or identical contributions) (Hetmank 2013; Rhyn and Blohm 2017), and of workers who do not actively participate in the exercise (e.g., through lack of time or attention). Intelligent mechanisms such as personalized nudging (Expert 2, 4, 5) can improve behavior or quality (Chiu et al. 2014; Haas et al. 2015; Riedl and Woolley 2017). One approach would be to use natural language understanding to automatically notify workers during the creation of a contribution that theirs is similar to other available contributions or is not sufficiently comprehensive (Case PSM) – for example, by displaying a uniqueness score (Expert 3).

*3) Operational Assistance.* When creating a contribution, workers may experience technical difficulties or develop questions regarding idea formulation (Adla et al. 2011; Hosseini et al. 2015). Usually, workers will either stop working on their contributions or contact the facilitator, who then has to step in and solve the problem (Adla et al. 2011), consuming the worker’s and the facilitator’s precious time. AI could identify the cause of either process- or technical-related problems and offer assistance. Missing information, which could hinder the workflow, could be identified by AI and provided at the appropriate time (Chittilappilly et al. 2016; Seeber et al. 2016). Robotic process automation (also referred to as intelligent automation technologies) could assign workers appropriate tasks based on the worker’s domain knowledge, previous crowdsourcing experience (Expert 4), or a lack of contributions in a specific task (Case PSM). Deep learning algorithms could help translate contributions or overcome language barriers (Expert 1). Pre-trained AI chat assistants could interactively explain the contribution creation process to the workers on a step-by-step basis and answer their questions accordingly (Tavanapour and Bittner 2018a).

*4) Workflow Enrichment.* To use the workers’ time as effectively as possible, facilitators break down the goal of an exercise into smaller tasks (Vukovic and Bartolini 2010). They efficiently integrate these tasks into an effective workflow supported by a crowdsourcing platform (Hetmank 2013). This is usually accompanied by a reduction in special attention to the needs of individual workers. AI could suggest the facilitator integrate additional or new information into the workflow (Chittilappilly et al. 2016; Riedl and Woolley 2017) or adjust the proposed next steps (Xiang et al. 2018). This could lead to a modified workflow or improved effectiveness. Natural language understanding could identify mismatches between the facilitator’s proposed task and the workers’ contributions, which may be the result of ambiguous task descriptions (Case PSM). Depending on the extent of the worker’s domain knowledge, the description of the task could be paraphrased or extended using natural language generation (Expert 1, 5). If workers do not find appropriate resources supporting their idea, natural language processing could identify the topic and the facilitator could then refer the worker to relevant data or scientific sources (Expert 6).

*5) Collaboration Guidance.* Facilitation, in a narrow sense, involves fostering collaboration and interdisciplinary exchange of information (Expert 2). However, such thematic exchange can go astray and move away from the exercise’s actual goal, despite facilitative support. Therefore, facilitators have to decide whether the existing discourse should be maintained or if the worker should be guided in a different direction (Xiang et al. 2018; Zheng et al. 2017). AI affords the evaluation of workers’ moods and the direction of the discussion with reference to the content. This provides the facilitator with a better understanding of the current atmosphere among the workers and the thematic focus of their collaboration. On the one hand, automated text mining, like sentiment detection, could generate semantic embeddings of the contributions (Expert 4) (Nagar et al. 2016), which could help to assess the maturity of the collaboration (Gimpel et al. 2020; Qiao et al. 2018). On the other hand, word-to-vec algorithms could focus the content of the discussion and uncover unprocessed areas (Expert 2, 3, 5) and frequently discussed topics (Case PSM), or help the facilitator to detect emerging topics (Case TCL).

*6) Worker Profiling.* Experienced facilitators mobilize the varied skills and expertise of the workers participating in an exercise (Tazzini et al. 2013). However, as the number of workers increases, getting to know one another becomes more difficult, particularly in an online crowdsourcing environment. Hence, facilitators may lack important information about workers, such as their previous experience in crowdsourcing or domain-specific skillsets. AI affords the use of interaction among workers (Dissanayake et al. 2014), as well as information on their backgrounds (Bozzon et al. 2013; Tazzini et al. 2013), to better assess the workers’ activity and the characteristics of their collaboration (Gimpel et al. 2020). Natural language generation could be used to process information from the worker’s publications or the worker’s social media profile to create a summary of the individual’s background (Expert 4). However, the rules of platform governance, as defined by the initiative stakeholders, must be upheld in any such investigations in order to avoid ethical concerns on the part of the workers (Alkharashi and Renaud 2018; Kocsis and Vreede 2016; Schlagwein et al. 2019). Alternatively, activity reports could be generated from the crowdsourcing platform via the use of (social) network algorithms (Case TCL) or natural language processing (Expert 1), leading to fully-automated dashboard generation for tracking the workers’ activity (Expert 6).

*7) Decision-making Preparation.* After one or more exercises, the workers will have provided several contributions. Facilitators then have to aggregate and synthesize these contributions into a meaningful foundation for decision-makers, such as a final report (Chan et al. 2016; Gimpel et al. 2020). AI affords support in decision-making preparation and could provide a synthesis, such as a decision template or recommendation for action to the requestors (Hetmank 2013) (Expert 2). Neural networks that have been specifically trained using vocabulary from the exercise’s domain could cluster the contributions and highlight unique ideas (Case TCL and PSM) (Expert 2, 5). Natural language understanding could be used to perform question answering based on the contributions, which could help a facilitator interact with the contributions and better understand the workers’ ideas, even after the exercise and without contacting the workers (Expert 3). Furthermore, summary generation algorithms could comprehensively synthesize the clustered contributions in ways that are meaningful for the decision-maker (Case PSM) (Expert 1, 2).

Despite the immense potential of AI in macro-task crowdsourcing facilitation, as reflected by the seven affordances, the interviewed experts stressed that researchers and facilitators must carefully consider which facilitation activity should be enabled or performed by AI (Expert 1, 2, 3, 4, 5). AI is prone to biases (Expert 4) and could systematically discriminate against specific workers (e.g., a natural language processing contribution evaluation algorithm could systematically down-rate contributions from workers with dyslexia). On top of that, the unreflected use of AI could lead facilitators to blindly believe in the underlying model and thereby reduce the overall level of goal achievement. Experts also argued that AI has limitations in understanding ethical and cultural factors and cannot fully imitate human interactions as facilitators (Expert 1, 5, 6). “I think it is really nice to have a name and a face to identify with a person who is communicating and asking you to do these things.” (Expert 5). Furthermore, facilitators should also consider the effort and difficulties during the development: “The art of AI is often not to solve the task, but to explain and teach the AI the task.” (Expert 3). Ultimately, the ill-considered use of AI in macro-task crowdsourcing could induce much bias in the outcome of an exercise (Expert 4) or decrease the workers’ participation and performance (Expert 6).

Even after a full review, we could not establish a hierarchy among the affordances. Nonetheless, an interlocking of individual affordances cannot be ruled out. To better illustrate the interdependencies of the affordances, the individual facilitation activities, and AI functions, Table 9 shows the revised version of the AI manifestation mapping. This table records all AI manifestations (i.e., specific action possibilities) that occurred during our research process. Every AI manifestation therein was observed either in literature (*L*), our observed crowdsourcing initiatives (*C*), or our interviews (*I*) and describes a possible shape of the corresponding affordance (*1)-7)*) concerning a facilitation activity or AI function.

**Table 9:** Revised Artificial Intelligence Manifestation Mapping

Facilitation Activity	Perceiving	Recognizing	Reasoning	Decision- making	Predicting	Generating	Acting
Contribution Evaluation		**1)** L C I	**1)** L I	**1)** L C I	**1)** L		**2)** L
Participation Encouragement							**2)** L C I
Worker Motivation							**2)** L C
Performance Monitoring		**6)** L C	**6)** L C I				**2)** L I
Quality Control		**6)** L I	**6)** L I		**6)** L C I		**2)** L C
Contribution Support						**3)** L C I	**3)** L I
Crowd Coordination		**3)** C		**3)** L I	**3)** L		**3)** L I
Task Communication			**4)** C				**4)** L I
Task Design				**4)** L I	**4)** L		**4)** L I
Tool Usage & Integration		**4)** I					
Workflow Design & Selection		**4)** L		**4)** L			
Crowd Moderation		**5)** L C I	**5)** L C I		**5)** L I		**5)** L I
Culture Development		**5)** I		**5)** I			**5)** L
Goal Orientation		**5)** L C I					**5)** L I
Risk Management		**5)** C I					**5)** L
Contribution Aggregation		**7)** L C I	**7)** L C I				**7)** L I
Decision Making						**7)** L C I	**7)** L
*Please note the following abbreviations*: *Affordances: (1) Contribution Assessment; (2) Improvement Triggering; (3) Operational Assistance; (4) Workflow Enrichment; (5) Collaboration Guidance; (6) Worker Profiling; (7) Decision-making Preparation* *Manifestations: L: observed in literature; C: observed in crowdsourcing initiative; I: observed in interviews*							

We observed that the way AI emerges in macro-task crowdsourcing facilitation is strongly dependent on the nature of the facilitation activity. In particular, we want to highlight two patterns in Table 9: Firstly, we did not find evidence for the AI function *perceiving* in any of the facilitation activities. We deem this to be due to the very nature of crowdsourcing initiatives since all data relevant to the facilitation of a crowdsourcing exercise has already been processed from the analog world (Hofmann et al. 2020). For instance, a conversation is not performed face-to-face but is stored in codified form on the crowdsourcing platform. Secondly, we argue that *culture development* is highly human-centered and requires empathy or social and emotional intelligence. Hence, there are very few cases wherein AI would have sufficient capabilities to perform this activity.

## Discussion

### Theoretical Contribution

In this research, we have addressed two research questions on the intersection of macro-task crowdsourcing, facilitation, and AI. To answer our research questions, our results encompass three novel theoretical contributions for scholars: a more precise understanding of macro-task crowdsourcing, an extensive list of 17 macro-task crowdsourcing facilitation activities, and seven holistic AI affordances in macro-task crowdsourcing.

Our work advances the domain of macro-task crowdsourcing by distinguishing macro-task crowdsourcing from other crowdsourcing types such as micro-task crowdsourcing or flash organizations. In doing so, it highlights the unique features of macro-task crowdsourcing, such as the low level of the problem’s decomposability and the nature of collaboration among workers that, together, form the demand for a facilitating instance. We further provide in-depth insights into two real-world macro-task crowdsourcing initiatives, including their particular AI tools, namely TCL and PSM. Both initiatives are dedicated to tackling wicked problems. Scholars can build upon this extensive understanding of macro-task crowdsourcing and better position their work in this area.

Our research contributes to the facilitation domain by deriving 17 macro-task crowdsourcing facilitation activities that holistically theorize facilitation as a suitable governance strategy for macro-task crowdsourcing. We also introduce a broad definition of macro-task crowdsourcing facilitation to merge the specific collaborative circumstances of macro-task crowdsourcing (Gimpel et al. 2020) with the current understanding of facilitation (Bostrom et al. 1993). This definition, along with the 17 activities, extends existing knowledge of facilitation and particular governance strategies for complex tasks in crowdsourcing, and may apply to other types of crowdsourcing or online collaboration. Our extensive understanding of facilitation in macro-task crowdsourcing differs from traditional knowledge of facilitation in that we consider the digital nature of crowdsourcing’s collaborations. With a more vital link to the crowd and increased attention to collaboration levels, our facilitation activities also extend existing crowdsourcing governance concepts more focused on the platform or the initiative. Fellow researchers can, for example, harness these activities as a starting point for further investigation in the context of crowdsourcing, which may be expanded over time and with technological advancement.

Finally, we use affordance theory as a socio-technical lens to extend the body of knowledge on AI-augmented facilitation. Our research identifies seven perceived AI affordances in macro-task crowdsourcing and generalized manifestations triangulated from practice, literature, and expertise. These manifestations were structured on seven abstract AI functions (Hofmann et al. 2020). Even though these could be seen to contrast to other (more technical) conceptualizations of AI, they performed well in the socio-technical context of macro-task crowdsourcing, describing how AI (could) occur within the 17 facilitation activities. Our affordances further extend these insights and holistically describe how AI can be applied by the facilitator in macro-task crowdsourcing facilitation. Through the insights from two macro-task crowdsourcing initiatives and six expert interviews, it is clearly demonstrated that AI currently holds only supportive potential for crowdsourcing. Although AI is now delivering super-human performances in some specific tasks, and while the digital starting conditions provided by crowdsourcing are promising, (digital) collaboration as an environment for AI is proving particularly challenging due to the subtle nuances of human interaction. However, we are convinced that AI’s potential will continue to increase as technologies evolve and will soon extend to collaboration and automation. Hence, we presume our AI affordances pave the way for AI scholars to undertake further research, for example, by helping scholars to structure future research projects or identify future research trends.

### Practical Implications

From a practical perspective, we see two major stakeholder groups benefiting from our research findings: AI developers and facilitators.

Firstly, developers of AI-augmented facilitation systems or functionalities can use our seven affordances as a starting point to identify areas for action or improvement and to implement innovative systems, tools, or functionalities that support facilitation in crowdsourcing. On top of this, our two macro-task crowdsourcing initiatives revealed good practices in which AI functionalities could add value to crowdsourcing exercises. AI developers could pick these insights and integrate AI functionalities, for example, into the crowdsourcing platform accordingly. Developers could also use the AI manifestation mapping to illustrate the status-quo in AI opportunities.

Secondly, the fact that current research on crowdsourcing facilitation lacks insights about AI means practitioners also stand to benefit from our results. With our seven developed and validated AI affordances, we provide guidance on which functionalities could add value when integrated before, during, or after crowdsourcing exercises. Thereby, our observed macro-task crowdsourcing initiatives and interviews with experts point out possible ways to include and integrate AI, which could be highly relevant when setting up new crowdsourcing initiatives. Furthermore, the 44 manifestations within our AI manifestation mapping provide initial indications of which AI functionalities or use-cases have already been considered and help facilitators correctly assess AI’s maturity. However, we recognize that the affordances are not equally relevant for all crowdsourcing exercises due to the complexity and variety of the latter. We argue that the list of affordances can also help communicate the usage and integration of AI tools or functionalities of existing crowdsourcing initiatives. This would also foster the exchange of knowledge in macro-task crowdsourcing, which is essential to find new approaches to tackling, for example, wicked problems in practice. Thereby, active and newly created crowdsourcing initiatives could increase their effectiveness and the efficiency of facilitation activities therein.

### Limitations and Future Research

Despite the comprehensive nature of our results, grounded in two literature searches and subject to a two-way practical validation with crowdsourcing initiatives and expert interviews, our research features some limitations in both the development and validation of the macro-task crowdsourcing facilitation activities and AI affordances.

The development of our 17 macro-task crowdsourcing activities was based on a structured literature search solely within the journal ‘Group Decision and Negotiation’. We deem its broad understanding of facilitation, developed over many years, to be sufficient for use in a crowdsourcing context. Nevertheless, we could have enhanced this literature search by including other journals or databases contributing to the crowdsourcing or facilitation domains.

Regarding the development of our AI research in macro-task crowdsourcing facilitation, we focused on creating perceived affordances. Due to the dynamic of the AI domain in crowdsourcing facilitation, we neither analyzed the actualization of their affordances nor elaborated on their existence (Ostern and Rosemann 2021; Volkoff and Strong 2017). Although we considered different forms of input (i.e., literature, crowdsourcing initiatives, expert interviews), we cannot formally claim that our affordances are complete. We argue that the fast-moving nature of research in AI could also impede efforts to compile an exhaustive list of AI affordances. Hence, increasing the scope of our literature searches could have resulted in a broader knowledge base. Furthermore, since we only analyzed macro-task crowdsourcing facilitation, we cannot verify the applicability or direct transferability to other types of crowdsourcing or collaboration. Testing whether our results can be generalized to other types of crowdsourcing and collaboration remains a task for future research.

Regarding the validation and refinement of our affordances, we only observed two macro-task crowdsourcing initiatives. We performed six interviews which, by their very nature, were both prone to bias (Yin 2018), such as response or selection bias. Even though we did not receive more insights at the end of our validation stage, we acknowledge that more initiatives or interviews could further improve or enhance our results. For instance, although we interviewed two experts with implementation knowledge, we can only make limited statements about the feasibility of implementing all affordances. Besides, even though experts have confirmed completeness, we still cannot guarantee that we have developed a complete list of affordances.

We hope that future research will address these limitations, offering multiple avenues for further investigation. Future research could specifically look into the practicability of the affordances. We anticipate that implementing AI-augmented facilitation prototypes for macro-task crowdsourcing based on our affordances will deliver valuable insights as to the feasibility of our affordances and their level of abstraction. Such prototypes could also enhance knowledge of actualized affordances or AI information systems in general and shed more light on how AI-augmented crowdsourcing could more efficiently tackle macro-tasks and their underlying complex problems. Furthermore, scholars could go beyond the descriptive nature of our results and (prescriptively) elaborate on how and why particular affordances enable macro-task crowdsourcing facilitation and how this could improve macro-task crowdsourcing initiatives. They also could use our affordances to extend design knowledge to design AI-augmented facilitation assistants (Maedche et al. 2019; Volkoff and Strong 2017). For example, researchers could derive design guidelines or principles that would aid facilitators in their burdensome amount of work (Gimpel et al. 2020).

## Conclusion

Our research was driven by opportunities that emerge from AI’s widespread application to aid facilitators. We turned to affordance theory to analyze AI’s potential applications in macro-task crowdsourcing facilitation. We answered our two research questions by defining macro-task crowdsourcing facilitation, constituting 17 activities, and introducing seven perceived AI affordances of macro-task crowdsourcing facilitation. We followed a two-stage, bottom-up approach consisting of an initial development stage comprising two literature searches, and a (second) validation & refinement stage, involving two macro-task crowdsourcing initiatives and six expert interviews. Our results could increase the efficiency of facilitation activities and the effectiveness of macro-task crowdsourcing, ultimately contributing to tackling wicked problems, such as the sustainable development goals.

## Figures and Tables

**Fig. 3:**
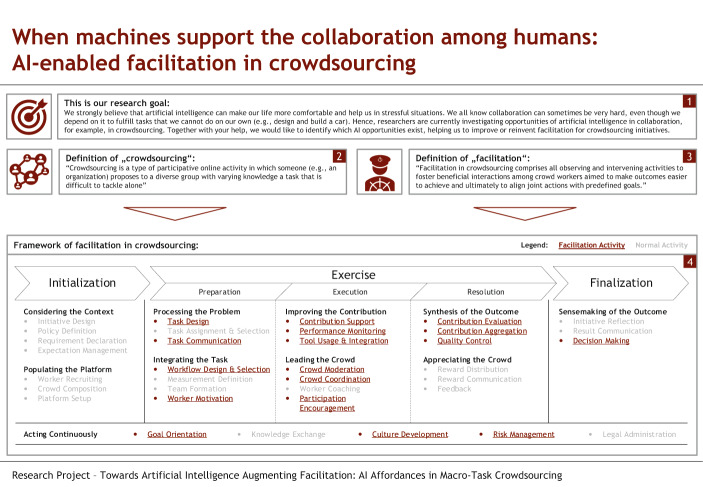
OnePager to Structure and Prepare the Interview Guide

**Fig. 4:**
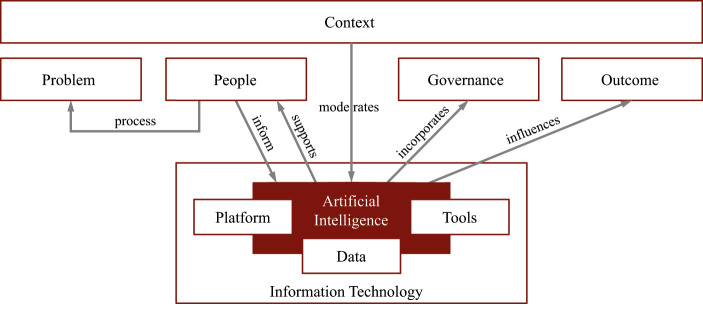
Visualization of Artificial Intelligence in Macro-Task Crowdsourcing

**Fig. 5:**
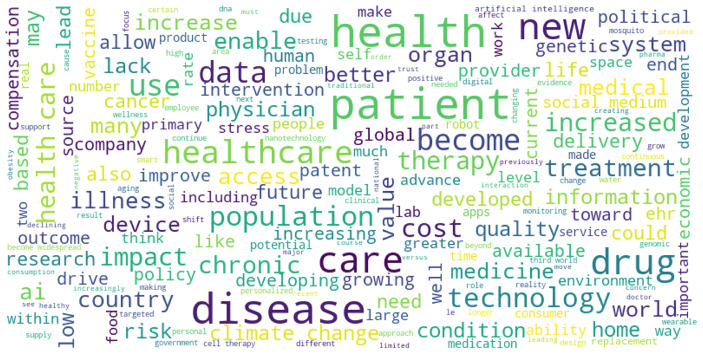
Word Cloud on All Contributions

**Fig. 6:**
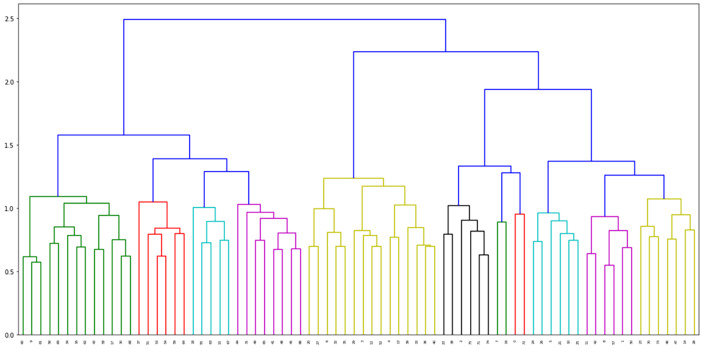
*Dendrogram of the Hierarchical Contribution Clustering*

**Fig. 7:**
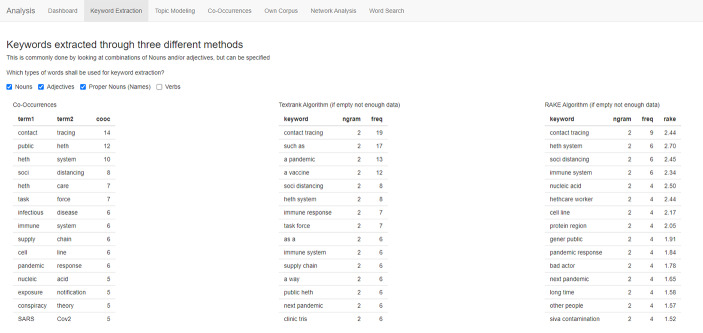
Keyword Extraction (With Permission of Lorenz Aull)

**Fig. 8:**
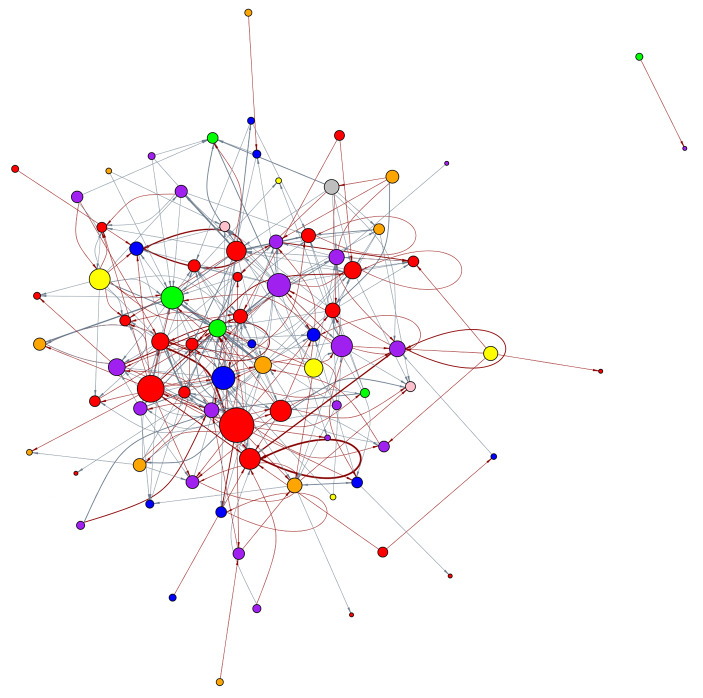
Anonymized Network Analysis of the Contributing Workers

## Data Availability

Not applicable.

## Appendix A - Literature Searches

### Appendix A.1 - Papers for Macro-Task Crowdsourcing Facilitation Activities

**Table 10:** Papers for Macro-Task Crowdsourcing Facilitation Activities

Authors	Title
Adla et al. (2011)	A Proposal of Toolkit for GDSS Facilitators
Antunes and Ho (2001)	The Design of a GDSS Meeting Preparation Tool
Azadegan and Kolfschoten (2014)	An Assessment Framework for Practicing Facilitator
Briggs et al. (2013)	Facilitator-in-a-Box: Process Support Applications to Help Practitioners Realize the Potential of Collaboration Technology
Franco and Nielsen (2018)	Examining Group Facilitation in Situ: The Use of Formulations in Facilitation Practice
Khalifa et al. (2002)	The Effects of Process and Content Facilitation Restrictiveness on GSS-Mediated Collaborative Learning
Kolfschoten et al. (2007)	Issues in the Design of Facilitated Collaboration Processes
Kolfschoten et al. (2011)	Modifiers for Quality Assurance in Group Facilitation
McCardle-Keurentjes and Rouwette (2018)	Asking Questions: A Sine Qua Non of Facilitation in Decision Support?
Vivacqua et al. (2011)	Computational Indicators to Assist Meeting Facilitation
de Vreede et al. (2002)	Towards an Instrument to Measure Participants’ Perceptions on Facilitation in Group Support Systems Meetings

### Appendix A.2 - Papers for Artificial Intelligence Manifestations

**Table 11:** Papers for Artificial Intelligence Manifestations

Authors	Title
Abhinav et al. (2018)	Crowdassistant: A Virtual Buddy for Crowd Worker
Gimpel et al. (2020)	Facilitating Like Darwin: Supporting Cross-Fertilisation in Crowdsourcing
Haas et al. (2015)	Argonaut: Macrotask Crowdsourcing for Complex Data Processing
Kittur et al. (2013)	The Future of Crowd Work
Nagar et al. (2016)	Accelerating the Review of Complex Intellectual Artifacts in Crowdsourced Innovation Challenges
Qiao et al. (2018)	Feedback Based High-Quality Task Assignment in Collaborative Crowdsourcing
Rhyn and Blohm (2017)	Combining Collective and Artificial Intelligence: Towards a Design Theory for Decision Support in Crowdsourcing
Rhyn et al. (2017)	Understanding the Emergence and Recombination of Distant Knowledge on Crowdsourcing Platforms
Seeber et al. (2016)	IT-Supported Formal Control: How Perceptual (In)Congruence Affects the Convergence of Crowd-Sourced Ideas
Seeber et al. (2020)	Machines as Teammates: A Research Agenda on AI in Team Collaboration
Tavanapour and Bittner (2018a)	Automated Facilitation for Idea Platforms: Design and Evaluation of a Chatbot Prototype
Tazzini et al. (2013)	A Structured Team Building Method for Collaborative Crowdsourcing

### Appendix A.3 - Assignments of the Found Statements About Artificial Intelligence

**Table 12:** Assignments of the Found Statements About Artificial Intelligence

Source	Statement	AI Function	Facilitation Activity
Abhinav et al. (2018)	Acts as a virtual buddy for the worker and helps them throughout their career journey	Acting	Worker Motivation
Abhinav et al. (2018)	Pro-actively supports worker’s needs	Acting	Contribution Support
Abhinav et al. (2018)	Data-driven advice to worker.	Generating	Contribution Support
Abhinav et al. (2018)	Helps the user to navigate crowdsourcing platform	Acting	Crowd Coordination
Abhinav et al. (2018)	Guides the user towards the most appropriate tasks	Acting	Goal Orientation
Abhinav et al. (2018)	Recommends the best career path progressions and skills to train in	Generating	Worker Motivation
Abhinav et al. (2018)	Recommends tasks to workers based on worker’s preference,	Generating	Task Communication
Abhinav et al. (2018)	Predicting fitment of a worker with the selected task, id est., how fit a worker is for the selected task	Predicting	Task Design
Abhinav et al. (2018)	Recommends the right marketplace to the crowd workers based on profile information	Generating	Task Communication
Abhinav et al. (2018)	Identify the goal of the worker	Recognizing	Goal Orientation
Abhinav et al. (2018)	Presents the response to the worker in a conversational interface.	Generating	Contribution Support
Abhinav et al. (2018)	Keeps track of the worker’s status and activities on the platform	Reasoning	Performance Monitoring
Gimpel et al. (2020)	Facilitator in achieving consensus within crowd discussions efficiently	Acting	Crowd Moderation
Gimpel et al. (2020)	Measuring and fostering cross-fertilization	Reasoning	Performance Monitoring
Gimpel et al. (2020)	Lead crowd discussions to better results	Acting	Crowd Moderation
Gimpel et al. (2020)	Facilitate online discussions	Reasoning	Crowd Moderation
Gimpel et al. (2020)	Decision support in crowdsourcing, yielding more efficient and effective decision-making.	Acting	Decision Making
Gimpel et al. (2020)	Track participants’ activity	Reasoning	Performance Monitoring
Gimpel et al. (2020)	Record activity data and calculate statistics by phase and type of activity per participant	Reasoning	Performance Monitoring
Gimpel et al. (2020)	Aggregate activity into relevant KPIs	Reasoning	Performance Monitoring
Gimpel et al. (2020)	Automatically extract activity data	Reasoning	Performance Monitoring
Gimpel et al. (2020)	Enrich activity statistics with background information	Recognizing	Performance Monitoring
Gimpel et al. (2020)	Assess the similarity between contributions	Reasoning	Quality Control
Gimpel et al. (2020)	Assess the semantic similarity of pairs or larger sets of contributions	Reasoning	Quality Control
Gimpel et al. (2020)	Provide a list of pairs/sets of potentially redundant contributions	Recognizing	Quality Control
Gimpel et al. (2020)	Provide a list of similar contributions	Recognizing	Quality Control
Gimpel et al. (2020)	Help participants when submitting their contributions to avoid redundancy	Acting	Contribution Support
Gimpel et al. (2020)	Group contributions which are thematically linked and identify the topics of these groups	Reasoning	Contribution Aggregation
Gimpel et al. (2020)	Provide suggestions of clusters, accounting for multiple levels of detail	Reasoning	Contribution Aggregation
Gimpel et al. (2020)	Provide suggestions for groups of contributions, which are easily rearrangeable	Reasoning	Contribution Aggregation
Gimpel et al. (2020)	Identify topic(s) in content groups	Recognizing	Contribution Aggregation
Gimpel et al. (2020)	Assess the knowledge domains captured by contributions	Reasoning	Contribution Evaluation
Gimpel et al. (2020)	Indicate the extent to which knowledge domains are represented per contribution	Reasoning	Contribution Evaluation
Gimpel et al. (2020)	Define a set of knowledge domains relevant to the given task	Predicting	Task Design
Gimpel et al. (2020)	Define a set of knowledge domains based on participants’ backgrounds	Predicting	Task Design
Gimpel et al. (2020)	Assess knowledge domains covered over time	Reasoning	Contribution Evaluation
Haas et al. (2015)	Predictive model of worker quality to select trusted workers to perform review	Predicting	Crowd Coordination
Haas et al. (2015)	A separate predictive model of task quality to decide which tasks to review	Predicting	Quality Control
Haas et al. (2015)	Identify the ideal trade-off between a single phase of review and multiple phases of review given a constrained review budget in order to maximize overall output quality	Reasoning	Workflow Design & Selection
Haas et al. (2015)	Reduce errors introduced by workers either unintentionally (due to innocent mistakes) or maliciously (due to collusion or spamming)	Recognizing	Quality Control
Haas et al. (2015)	Organizes the crowd hierarchically	Acting	Crowd Coordination
Haas et al. (2015)	Provides a predictive model of task error	Predicting	Quality Control
Haas et al. (2015)	Tracks worker quality over time	Reasoning	Performance Monitoring
Haas et al. (2015)	Promote the most qualified workers to the top of the hierarchy	Decision-making	Worker Motivation
Haas et al. (2015)	Selecting tasks to review	Recognizing	Quality Control
Haas et al. (2015)	Identify skilled workers	Recognizing	Participation Encouragement
Haas et al. (2015)	Determining the quality of a task	Predicting	Task Design
Kittur et al. (2013)	Tasks may be structured through multi-stage workflows in which workers may collaborate either synchronously or asynchronously. As part of this, AI may guide (and be guided by) crowd workers.	Acting	Crowd Coordination
Kittur et al. (2013)	Decomposing tasks into subtasks	Acting	Task Design
Kittur et al. (2013)	Assignment of tasks in relation to individuals’ abilities	Decision-making	Task Design
Kittur et al. (2013)	Guide workers to complete synchronous tasks	Acting	Contribution Support
Kittur et al. (2013)	Automatic assignment of group members to maximize collective intelligence	Decision-making	Task Design
Kittur et al. (2013)	Help make the crowd more efficient, skilled, and accurate	Acting	Crowd Coordination
Kittur et al. (2013)	Design machine learning algorithms that more deeply understand the human nature of these labels	Reasoning	Contribution Evaluation
Kittur et al. (2013)	Determine which work products may still be improved	Recognizing	Quality Control
Kittur et al. (2013)	Assign workers most likely to make such improvements	Decision-making	Task Design
Kittur et al. (2013)	Predict their expertise needs in advance, then train and adapt workers in an online fashion via automated tutoring or peer learning	Predicting	Task Design
Kittur et al. (2013)	Designing and integrating workflow, incentive, and instruction patterns	Decision-making	Workflow Design & Selection
Kittur et al. (2013)	Serve as a reflective aids, encouraging the crowd to learn by pointing out what others have done in similar contexts	Acting	Crowd Moderation
Kittur et al. (2013)	Sharing information about workers should be coupled with more robust systems for monitoring and reporting requester abuses	Reasoning	Performance Monitoring
Kittur et al. (2013)	Guiding crowds on which tasks to complete (task assignment)	Decision-making	Crowd Coordination
Nagar et al. (2016)	Reduce the cognitive load of expert judges	Acting	Contribution Evaluation
Nagar et al. (2016)	Classify and rate crowd-proposals	Reasoning	Contribution Evaluation
Nagar et al. (2016)	Indicators of the completeness and maturity of the proposal	Reasoning	Contribution Evaluation
Nagar et al. (2016)	Organize the review process somewhat differently, and hopefully, more efficiently	Decision-making	Workflow Design & Selection
Nagar et al. (2016)	Prioritize the review sequence	Decision-making	Contribution Evaluation
Nagar et al. (2016)	Automatically scoring these complex intellectual artifacts	Reasoning	Quality Control
Nagar et al. (2016)	Aid human experts in the review process	Acting	Contribution Evaluation
Qiao et al. (2018)	Distributes the skillful workers and less-skilled workers	Decision-making	Task Design
Qiao et al. (2018)	Determine which worker or group of workers should be assigned tasks	Recognizing	Task Design
Qiao et al. (2018)	Records everyone’s work execution time	Reasoning	Performance Monitoring
Qiao et al. (2018)	Maximizes the overall assignment quality	Acting	Task Design
Rhyn and Blohm (2017)	Extract useful information from unstructured data	Recognizing	Contribution Evaluation
Rhyn and Blohm (2017)	Cluster ideas	Reasoning	Contribution Aggregation
Rhyn and Blohm (2017)	Selecting novel ideas	Recognizing	Contribution Evaluation
Rhyn and Blohm (2017)	A DSS should identify relevant contributions in the data set.	Recognizing	Contribution Evaluation
Rhyn and Blohm (2017)	A DSS should remove irrelevant contributions from the data set.	Acting	Contribution Aggregation
Rhyn and Blohm (2017)	A DSS should aggregate redundant information for the decision-maker.	Reasoning	Contribution Aggregation
Rhyn and Blohm (2017)	A DSS should prioritize important information for the decision-maker.	Decision-making	Decision Making
Rhyn et al. (2017)	Tracking the origin of contributions in crowdsourcing	Reasoning	Contribution Evaluation
Rhyn et al. (2017)	Analyzing their textual characteristics	Reasoning	Quality Control
Rhyn et al. (2017)	Identifying the most innovative ones	Recognizing	Contribution Evaluation
Rhyn et al. (2017)	Predictors for innovative contributions	Predicting	Contribution Evaluation
Rhyn et al. (2017)	Evaluation of large amounts of contributions	Reasoning	Contribution Evaluation
Seeber et al. (2016)	Facilitate the development of shared understanding	Acting	Crowd Moderation
Seeber et al. (2016)	Improved idea quality	Acting	Quality Control
Seeber et al. (2016)	Recommendations could be designed that provide feedforward guidance to extend idea descriptions	Predicting	Crowd Moderation
Seeber et al. (2020)	Automatically guide the behavior of humans, such as imposing communication patterns onto the group, asking clarification questions, giving recommendations, or providing feedback	Acting	Crowd Moderation
Seeber et al. (2020)	Helps evaluate the consequences of potential solutions	Acting	Contribution Evaluation
Seeber et al. (2020)	Debates the validity of proposed positions offering evidence and arguments	Generating	Decision Making
Seeber et al. (2020)	Provides predictions to unstructured problems	Predicting	Task Design
Seeber et al. (2020)	Participates in cognitive decision making with human actors	Acting	Decision Making
Seeber et al. (2020)	Incorporate and understand emotional signals from humans	Reasoning	Crowd Moderation
Seeber et al. (2020)	Support the team in coming up with conclusions	Acting	Contribution Support
Seeber et al. (2020)	Identify certain group dynamics	Recognizing	Crowd Moderation
Seeber et al. (2020)	Foster team cohesion	Acting	Culture Development
Seeber et al. (2020)	Mitigating negative cognitive biases	Acting	Risk Management
Tavanapour and Bittner (2018)	Facilitation of the idea submission process	Acting	Contribution Support
Tavanapour and Bittner (2018)	Reach each contributor on idea platforms in an initial idea submission process	Acting	Task Communication
Tavanapour and Bittner (2018)	Actively asks questions in one-to-one collaboration and encourages the contributor to think about missing details and add them before the idea is released to the filtering or voting process	Acting	Quality Control
Tavanapour and Bittner (2018)	Guide users in such a way that they can more thoroughly elaborate on their ideas	Acting	Crowd Moderation
Tavanapour and Bittner (2018)	React correctly to statements	Acting	Crowd Moderation
Tavanapour and Bittner (2018)	Actively ask questions	Generating	Crowd Moderation
Tavanapour and Bittner (2018)	Lead conversation	Acting	Crowd Moderation
Tavanapour and Bittner (2018)	Motivate participants to qualitatively edit initial ideas	Acting	Participation Encouragement
Tavanapour and Bittner (2018)	Display productivity oriented behavior	Reasoning	Performance Monitoring
Tavanapour and Bittner (2018)	Prevent deviation to other topics & guide conversation back to idea	Acting	Goal Orientation
Tavanapour and Bittner (2018)	Construct interesting & pleasant conversation	Generating	Crowd Moderation
Tavanapour and Bittner (2018)	Provide correct reactions to statements	Generating	Crowd Moderation
Tazzini et al. (2013)	Favoring motivation and creative participation among users	Acting	Participation Encouragement
Tazzini et al. (2013)	Track and quantify the contribution of each solver to the final solution	Reasoning	Performance Monitoring
Tazzini et al. (2013)	Boosting motivation	Acting	Worker Motivation
Tazzini et al. (2013)	Supporting and driving users creativity	Acting	Contribution Support
Tazzini et al. (2013)	Ensures fairness and objectivity in measuring the contribution of each individual	Acting	Performance Monitoring
Tazzini et al. (2013)	Select the experts with the required skills for the specific submitted problem	Decision-making	Task Design
Tazzini et al. (2013)	Dynamic evaluation of individuals’ ability	Reasoning	Task Design

## Appendix B - Refinement of the AI Manifestation Mapping

During the analysis of TCL, PSM, and their corresponding platforms, we derived 29 potential use-cases of AI-augmented facilitation, of which 10 have been actualized and 19 have been perceived as helpful to facilitate crowdsourcing initiatives. These 29 potential use-cases resulted in 18 distinct manifestations using the same matching approach as in step II). Three manifestations were not found within our literature search. Thus, we extended our AI manifestation mapping (i.e., recognizing, in crowd coordination; recognizing, in risk management; reasoning, in task communication). These three manifestations could all be assigned to the existing seven affordances. Hence, it was not necessary to change the affordances. Besides the potential use-cases and manifestations, we observed that the timing of the decision to use AI impacted the potential for integration. In TCL, the decision to include AI was made following our recognition that the workers had created many contributions during an exercise. Hence, considerations about the use of AI revolved around aggregating the contributions, which can be seen as the affordance 7) decision-making preparation. In PSM, a dedicated AI team had been set up during the workflow design, which specifically developed an extensive set of AI functionalities for facilitators, including use-cases of 6) worker profiling and 5) collaboration guidance. We conclude that, if facilitators are aware of AIs’ potential before the start of the exercise, they can derive more benefits from it.

In step V) Interviews, we conducted six expert interviews to validate and refine the AI manifestation mapping and the initial AI affordances. We integrated the input we received into the affordances after each interview and later analyzed the content in a structured manner in line with Schreier (2012). We gathered 72 statements about potential use-cases for AI in facilitation, which led to 30 distinct manifestations. Three manifestations were not identified by literature or the case studies (i.e., recognizing in culture development, decision-making in culture development, and recognizing in tool usage & integration). We were able to assign these three new manifestations to our affordances.

## Appendix C - Interviews

### Appendix C.1 - Interview Guide

Ideation

1. Can you tell me in three or four sentences about your previous experience with *Artificial Intelligence or Crowdsourcing Facilitation?*
2. Have you ever experienced the use of *Artificial Intelligence* in collaboration for facilitation purposes in general?
3. How do you imagine *Artificial Intelligence* affecting *Crowdsourcing Facilitation?*
4. Imagine the following situation: We both are organizing a crowdsourcing exercise with about 200 experts. Our capacity regarding facilitators is not limited. So we can let the facilitators do whatever we want. What activities would you let these facilitators do in order to maximize the value of the crowdsourcing exercise?

Detailed Feedback

1. In box "4" of our OnePager *(see Figure 3)*, we depicted a framework of crowdsourcing from a processual perspective.
  - Where do you see the most significant impact of AI in this figure?
  - Do you see any of these activities that can not be supported or enabled by AI?

2. Please take a moment to read through the table on page 2 *(reference was made to the respective status of the AI affordances - see Table 6)*. These are AI opportunities in crowdsourcing that we found in the literature.
  - What do you think about them?
  - How comprehensible do you find this list?
  - To what extent do you believe that these AI opportunities are feasible or applicable in today’s crowdsourcing exercises?
  - What is missing?
3. *(This part was added after the first two interviews)* Please take a look at page 3 *(reference was made to the AI manifestations as well as a graphical representation of AI with-in macro-task crowdsourcing - see Table 7 and Figure 4)*. These are two different illustrations that try to shed more light on how artificial intelligence influences crowdsourcing.
  - Which illustration is more appealing to you and why?
  - What could be improved in the illustration you prefer to make it more informative?

### Appendix C.2 - Validation of the Artificial Intelligence Affordances

We validated our affordances according to four defined criteria: completeness, comprehensiveness, meaningfulness, level of detail, and applicability in today’s crowdsourcing initiatives (Sonnenberg and vom Brocke 2012). We could confirm four affordances without changes to their description or name (i.e., contribution assessment, operational assistance, collaboration guidance, and decision-making preparation). We could also confirm two further affordances with changes to their description (i.e., improvement triggering and worker profiling). For 2) improvement triggering experts highlighted that low activity does not necessarily mean that a worker should be considered “bad” (Expert 2, 6) and that it is essential to “understand why underperforming is happening” (Expert 5). Since the other experts see AI as capable of identifying and nudging inactive workers or triggering improvements (Expert 1, 3, 4), we enhanced the description of the affordance by including the identification of non-active workers. Experts (Expert 2, 4) specifically noted that including information about workers’ backgrounds could be beneficial for monitoring the performance and moderating the crowd. Even though we agree with Expert 1 that facilitators have to consider ethical standards when profiling workers, we see benefits in considering the workers’ skills (e.g., a better understanding of the workers’ communication network or identifying echo chambers) and so changed the description of 6) worker profiling. Finally, we could confirm 4) workflow enrichment after substantially and successively revising the name (previously: environment creation) and the affordance description. Experts felt that the previous affordance description was not comprehensive enough (Expert 1, 2), too broad (Expert 4, 6), or not applicable to crowdsourcing initiatives (Expert 2, 3). We also picked up the feedback that the intended affordance is still mainly human-driven (Expert 2) and that AI could enrich the exercise with relevant information regarding the workflow or the discussed content (Expert 1, 3, 4). Hence, we shifted the focus of this affordance from creating and maintaining a highly productive collaboration and communication environment to providing and integrating useful information and knowledge into the predefined workflow, which was perceived as meaningful (Experts 5, 6)

In addition to these suggestions for improvement, experts stated that there was no relevant aspect missing (Expert 2, 3, 4, 5, 6), from which we concluded that our affordances were complete. Five of the experts explicitly confirmed the comprehensiveness of the name and the description of the affordances (Expert 2, 3, 4, 5, 6). Expert 5 specifically stated, “I think it is the first time I had a research interview where there had been this comprehensible material for me to look at.” According to all of the interviewed experts, our affordances are meaningful. Even though the affordances are not mutually exclusive and show some differences in the granularity of formulation, the experts did not see any problems regarding the level of detail (Expert 2). The experts also explicitly acknowledged the applicability of specific affordances in today’s crowdsourcing initiatives (Expert 1, 2, 3, 4, 6)

### Appendix C.3 - Empirical Evidence From the Interviews

**Table 13:** Powerful Quotes From Within the Interviews (* translated to English)

ID	Affordance Name	Exemplary Quotes From the Experts
**1)**	Contribution Assessment	*Expert 3**: “And it can be very useful to find similar discussion threads and similar ideas.” *Expert 5*: “Understand all the results from a crowdsourcing exercise in a way that’s empirical and meaningful.” *Expert 6*: “There is also all of this other work [besides creating the contributions] that[’s analyzation] would help with the facilitation.”
**2)**	Improvement Triggering	*Expert 2**: “So maybe also a little bit set a spark, a stimulus, or a nudge.” *Expert 4**: “What a moderator often does is triggering. […] So I try to inspire them [the worker] or give them some guidance. So that they can do it, and that’s where I think you can use AI again.” *Expert 5*: “Obviously, you could have automated message’s that encourage people to participate if they haven’t done something on the site.”
**3)**	Operational Assistance	*Expert 1**: “The second thing that I would find exciting are intelligent translation programs. […] To use them in such a way that language barriers are broken down to some extent.” *Expert 3**: “For example, you could show a novelty score to the user as they type in their idea.” *Expert 5*: “So one way I could see that happening: if people happen submitting ideas using the same syntax or verbiage, then a tool could be like ‘Oh hey, maybe you would want to work with this other individual submitting similar ideas.’”
**4)**	Workflow Enrichment	*Expert 3**: “What has a lot of value for me personally with AI and data, which is probably not the case with facilitators, is ‘how do we now, as the main facilitator, transfer these learnings into the next task?’.” *Expert 5*: “Tool usage and integration, I think, could also be something that a tool could do. So, for example, it could say, ‘You haven’t done this yet. Maybe you want to check this feature out.’ or something like that.” *Expert 5*: “Communicating the task is something that I see being pre-generated, and humans go in and do checks and make sure that it is okay.”
**5)**	Collaboration Guidance	*Expert 3** “In that respect, for crowd moderation or crowd coordination, Word-to-Vec would be great because you have a neutral algorithm that just shows what’s happening without understanding whether that’s good, bad, or whatever.” *Expert 4** “When it comes to, ‘Tell me what’s relevant here in this data set.’ that I could just say, ‘Look, could this be relevant here?’ and I sort of let it [the AI] do the assessment after that.” *Expert 6*: “I think something that could be helpful here, regarding contribution development, is if people find proposals that are similar or related to the ones that they are working on.”
**6)**	Worker Profiling	*Expert 1**: “And of course, what is based on numbers or is evaluable. In the sense that you say quality control should depend on the number of contributions or spelling. These are things that systems like NLP or machine learning can test well. Here you can set thresholds creating a warning: ‘Attention [contribution] quality is decreasing’.” *Expert 2**: “One activity was redundancy detection. So, we wanted to avoid contributions are too similar.” *Expert 4**: Well, you could do profiling of people. You could do a priori profiling. You take their CVs, LinkedIn profiles, and maybe their publications or articles and profile them based on that.
**7)**	Decision- making Preparation	*Expert 1**: “And of course, everything goes in the direction of aggregating and interpreting results. Some technologies create natural text, that is, to create reports from the contributions. So clustering is somehow an exciting topic.” *Expert 2**: “I see the use of AI more in the sense of massive automation, simply getting information much faster and then ultimately providing the facilitator with a decision template, preferably also with recommendations for action.” *Expert 4**: “Also something like search, if you are very specifically interested in: ‘How do people think about electric cars?’. […] Today, AI methods have transferred semantic text understanding to machines.”

## References

[CR1] Abhinav K, Dubey A, Jain S, Bhatia GK, McCartin B, Bhardwaj N (2018) “Crowdassistant: a virtual buddy for crowd worker,” in *Proceedings of the 5th International Workshop on Crowd Sourcing in Software Engineering*, pp. 17–20 (doi: 10.1145/3195863.3195865)

[CR2] Achmat L, Brown I (2019) “Artificial intelligence affordances for business innovation: a systematic review of literature,” in *Proceedings of 4th International Conference on the Internet, Cyber Security and Information Systems*, pp. 1–12

[CR3] Adla A, Zarate P, Soubie J-L (2011). A proposal of toolkit for GDSS facilitators. Group Decis Negot.

[CR4] Alabduljabbar R, Al-Dossari H (2016) “A Task Ontology-based Model for Quality Control in Crowdsourcing Systems,” in *Proceedings of the International Conference on Research in Adaptive and Convergent Systems*, pp. 22–28 (doi: 10.1145/2987386.2987413)

[CR5] Alford J, Head BW (2017). Wicked and less wicked problems: a typology and a contingency framework. Policy and Society.

[CR6] Alkharashi A, Renaud K (2018) “Privacy in crowdsourcing: a systematic review,” in *ISC 2018: Information Security*, pp. 387–400 (doi: 10.1007/978-3-319-99136-8_21)

[CR7] Alsheibani S, Cheung Y, Messom C (2018) “Artificial intelligence adoption: AI-readiness at firm-level,” in *Proceedings of the 22nd Pacific Asia Conference on Information Systems (PACIS 2018)*, Association for Information Systems

[CR8] Antunes P, Ho T (2001). The design of a GDSS meeting preparation tool. Group Decis Negot.

[CR9] Asatiani A, Malo P, Nagbøl PR, Penttinen E, Rinta-Kahila T, Salovaara A (2021). Sociotechnical Envelopment of Artificial Intelligence: An Approach to Organizational Deployment of Inscrutable Artificial Intelligence Systems. J Association Inform Syst.

[CR10] Askay D (2017) “A conceptual framework for investigating organizational control and resistance in crowd-based platforms,” in *Proceedings of the 50th Hawaii International Conference on System Sciences (HICSS 2017)*

[CR11] Assis Neto FR, Santos CAS (2018). Understanding crowdsourcing projects: A systematic review of tendencies, workflow, and quality management. Inf Process Manag.

[CR12] Autio E, Nambisan S, Thomas LDW, Wright M (2018). Digital affordances, spatial affordances, and the genesis of entrepreneurial ecosystems. Strateg Entrepreneurship J.

[CR13] Azadegan A, Kolfschoten G (2014). An assessment framework for practicing facilitator. Group Decis Negot.

[CR14] Bawack RE, Wamba F, Carillo KDA (2019) “Artificial intelligence in practice: implications for IS research,” in *Proceedings of the 25th Americas Conference on Information Systems (AMCIS 2019)*, Association for Information Systems

[CR15] Bayer S, Gimpel H, Rau D (2020). IoT-commerce - opportunities for customers through an affordance lens. Electron Markets.

[CR16] Belleflamme P, Lambert T, Schwienbacher A (2014). Crowdfunding: Tapping the right crowd. J Bus Ventur.

[CR17] Benbya H, Pachidi S, Jarvenpaa S (2021). Special Issue Editorial: Artificial Intelligence in Organizations: Implications for Information Systems Research. J Association Inform Syst.

[CR18] Blohm I, Leimeister JM, Krcmar H (2013). Crowdsourcing: how to benefit from (too) many great ideas. MIS Q Exec.

[CR19] Blohm I, Zogaj S, Bretschneider U, Leimeister JM (2018). How to manage crowdsourcing platforms effectively?. Calif Manag Rev.

[CR20] Blohm I, Zogaj S, Bretschneider U, Leimeister JM (2020). How to Manage Crowdsourcing Platforms Effectively. NIM Mark Intell Rev.

[CR21] Bogers M, Chesbrough H, Moedas C (2018). Open Innovation: Research, Practices, and Policies. Calif Manag Rev.

[CR22] Bostrom RP, Anson R, Clawson VK(1993) “Group facilitation and group support systems,”Group support systems: New perspectives(8), pp.146–168

[CR23] Boughzala I, de Vreede T, Nguyen C, Vreede G-Jde (2014) “Towards a Maturity Model for the Assessment of Ideation in Crowdsourcing Projects,” in *Proceedings of the 47th Hawaii International Conference on System Sciences (HICSS 2014)*, pp. 483–490 (doi: 10.1109/HICSS.2014.67)

[CR24] Bozzon A, Brambilla M, Ceri S, Silvestri M, Vesci G (2013) “Choosing the right crowd: expert finding in social networks,” in *Proceedings of the 16th International Conference on Extending Database Technology*, pp. 637–648 (doi: 10.1145/2452376.2452451)

[CR25] Briggs RO, Kolfschoten GL, de Vreede G-J, Lukosch S, Albrecht CC (2013). Facilitator-in-a-box: process support applications to help practitioners realize the potential of collaboration technology. J Manage Inform Syst.

[CR26] Bruno JF, Stachowicz JJ, Bertness MD (2003). Inclusion of facilitation into ecological theory. Trends Ecol Evol.

[CR27] Brynjolfsson E, McAffe A(2017) “The business of artificial intelligence,”Harvard Business Review, pp.1–20

[CR28] Brynjolfsson E, Rock D, Syverson C (2017) “Artificial intelligence and the modern productivity paradox: a clash of expectations and statistics. ” National Bureau of Economic Research

[CR29] Burlamaqui L, Dong A (2015) “The use and misuse of the concept of affordance,” in *Design Computing and Cognition*, pp. 295–311 (doi: 10.1007/978-3-319-14956-1_17)

[CR30] Cer D, Yang Y, Kong S, Hua N, Limtiaco N, Constant R, Guajardo-Cespedes N, Yuan M, Tar S, Sung C, Strope Y-H, Kurzweil R (2018) “Universal Sentence Encoder,&#8221

[CR31] Chan J, Dang S, Dow SP (2016) “Improving Crowd Innovation with Expert Facilitation,” in *Proceedings of the 19th ACM Conference on Computer-Supported Cooperative Work & Social Computing*, pp. 1223–1235 (doi: 10.1145/2818048.2820023)

[CR32] Chittilappilly AI, Chen L, Amer-Yahia S (2016). A survey of general-purpose crowdsourcing techniques. IEEE Trans Knowl Data Eng.

[CR33] Chiu C-M, Liang T-P, Turban E (2014). What can crowdsourcing do for decision support?. Decis Support Syst.

[CR34] Clawson VK, Bostrom RP (1996). Research-driven facilitation training for computer-supported environments. Group Decis Negot.

[CR35] Cullina E, Conboy K, Morgan L (2015) “Measuring the crowd: a preliminary taxonomy of crowdsourcing metrics,” in *Proceedings of the 11th International Symposium on Open Collaboration* (doi: 10.1145/2788993.2789841)

[CR36] de Vreede G-J, Briggs R (2018) “Collaboration engineering: reflections on 15 years of research & practice,” in *Proceedings of the 51st Hawaii International Conference on System Sciences (HICSS 2018)*

[CR37] de Vreede G-J, Briggs RO (2019). A program of collaboration engineering research and practice: contributions, insights, and future directions. J Manage Inform Syst.

[CR38] de Vreede G-J, Niederman F, Paarlberg I (2002). Towards an instrument to measure participants’ perceptions on facilitation in group support systems meetings. Group Decis Negot.

[CR39] de Vreede T, Steele L, de Vreede G-J, Briggs R (2020) “LeadLets: Towards a Pattern Language for Leadership Development of Human and AI Agents,” in *Proceedings of the 53rd Hawaii International Conference on System Sciences*, T. Bui (ed.), Hawaii International Conference on System Sciences (doi: 10.24251/HICSS.2020.084)

[CR40] Derrick DC, Read A, Nguyen C, Callens A, de Vreede G-J (2013) “Automated group facilitation for gathering wide audience end-user requirements,” in *Proceedings of the 46th Hawaii International Conference on System Sciences (HICSS 2013)*, pp. 195–204 (doi: 10.1109/HICSS.2013.109)

[CR41] Dissanayake I, Nerur S, Zhang J (2019) “Team formation and performance in online crowdsourcing competitions: the role of homophily and diversity in solver characteristics,” in *Proceedings of the 40th International Conference on Information Systems (ICIS 2019)*, Association for Information Systems

[CR42] Dissanayake I, Zhang J, Gu B (2014) “Virtual team performance in crowdsourcing contests: a social network perspective,” in *Proceedings of the 35th International Conference on Information Systems (ICIS 2014)*, Association for Information Systems

[CR43] Dissanayake I, Zhang J, Gu B (2015). Task division for team success in crowdsourcing contests: resource allocation and alignment effects. J Manage Inform Syst.

[CR44] Dissanayake I, Zhang J, Yuan F, Wang J (2015b) “Peer-recognition and performance in online crowdsourcing communities,” in *Proceedings of the 48th Hawaii International Conference on System Sciences (HICSS 2015)*, pp. 4262–4265 (doi: 10.1109/HICSS.2015.646)

[CR45] Dremel C, Herterich MM, Wulf J, vom Brocke J (2020). Actualizing big data analytics affordances: a revelatory case study. Inf Manag.

[CR46] Du W, Pan SL, Leidner DE, Ying W (2019). Affordances, experimentation and actualization of FinTech: a blockchain implementation study. J Strateg Inf Syst.

[CR47] Dwivedi YK, Hughes L, Ismagilova E, Aarts G, Coombs C, Crick T, Duan Y, Dwivedi R, Edwards J, Eirug A, Galanos V, Ilavarasan PV, Janssen M, Jones P, Kar AK, Kizgin H, Kronemann B, Lal B, Lucini B, Medaglia R, Le Meunier-FitzHugh K, Le Meunier-FitzHugh LC, Misra S, Mogaji E, Sharma SK, Singh JB, Raghavan V, Raman R, Rana NP, Samothrakis S, Spencer J, Tamilmani K, Tubadji A, Walton P, Williams MD (2021). Artificial Intelligence (AI): Multidisciplinary perspectives on emerging challenges, opportunities, and agenda for research, practice and policy. Int J Inf Manag.

[CR48] Erickson LB, Petrick I, Trauth EM (2012) “Organizational Uses of the Crowd: Developing a Framework for the Study of Crowdsourcing,” in *Proceedings of the 50th Annual Conference on Computers and People Research*, New York, NY, USA: ACM, pp. 155–158 (doi: 10.1145/2214091.2214133)

[CR49] Estellés-Arolas E, González-Ladrón-de-Guevara F (2012). Towards an integrated crowdsourcing definition. J Inform Sci.

[CR50] Faik I, Barrett M, Oborn E (2020). How Information Technology Matters in Societal Change: An Affordance-Based Institutional Perspective. MIS Q.

[CR51] Faullant R, Dolfus G (2017) “Everything community? Destructive processes in communities of crowdsourcing competitions,” *Business Process Management Journal* (23:6, SI), pp. 1108–1128 (doi: 10.1108/BPMJ-10-2016-0206)

[CR52] Franco LA, Nielsen MF (2018). Examining group facilitation in situ: the use of formulations in facilitation practice. Group Decis Negot.

[CR53] Fritz S, See L, Carlson T, Haklay M, Oliver JL, Fraisl D, Mondardini R, Brocklehurst M, Shanley LA, Schade S, Wehn U, Abrate T, Anstee J, Arnold S, Billot M, Campbell J, Espey J, Gold M, Hager G, He S, Hepburn L, Hsu A, Long D, Masó J, McCallum I, Muniafu M, Moorthy I, Obersteiner M, Parker AJ, Weisspflug M, West S (2019). Citizen science and the United Nations Sustainable Development Goals. Nat Sustain.

[CR54] Füller J, Hutter K, Kröger N (2021). Crowdsourcing as a service – from pilot projects to sustainable innovation routines. Int J Project Manage.

[CR55] Gaver WW (1991) “Technology affordances,” in *Proceedings of the SIGCHI conference on Human factors in computing systems Reaching through technology*, S. P. Robertson, G. M. Olson and J. S. Olson (eds.), pp. 79–84 (doi: 10.1145/108844.108856)

[CR56] Geiger D, Schader M (2014). Personalized task recommendation in crowdsourcing information systems — Current state of the art. Decis Support Syst.

[CR57] Geiger D, Seedorf S, Schulze T, Nickerson RC, Schader M (2011) “Managing the Crowd: Towards a Taxonomy of Crowdsourcing Processes,” in *Proceedings of the 17th Americas Conference on Information Systems (AMCIS 2011)*, Association for Information Systems

[CR58] Ghezzi A, Gabelloni D, Martini A, Natalicchio A (2018). Crowdsourcing: a review and suggestions for future research. Int J Manage Reviews.

[CR59] Gibson JJ (1977). The theory of affordances. Hilldale USA.

[CR60] Gimpel H, Graf-Drasch V, Laubacher RJ, Wöhl M (2020). Facilitating like Darwin: supporting cross-fertilisation in crowdsourcing. Decis Support Syst.

[CR61] Griffith TL, Sawyer JE, Poole MS (2019). Systems Savvy: Practical Intelligence for Transformation of Sociotechnical Systems. Group Decis Negot.

[CR62] Haas D, Ansel J, Gu L, Marcus A (2015) “Argonaut: macrotask crowdsourcing for complex data processing,” *Proceedings of the VLDB Endowment* (8:12), pp. 1642–1653 (doi: 10.14778/2824032.2824062)

[CR63] Haenlein M, Kaplan A (2019). A Brief History of Artificial Intelligence: On the Past, Present, and Future of Artificial Intelligence. Calif Manag Rev.

[CR64] Head BW, Alford J (2015). Wicked problems: implications for public policy and management. Adm Soc.

[CR65] Hetmank L (2013) “Components and functions of crowdsourcing systems - a systematic literature review,” in *Wirtschaftsinformatik Proceedings (WI 2013)*, p. 2013

[CR66] Hinsen S, Hofmann P, Jöhnk J, Urbach N (2022) “How Can Organizations Design Purposeful Human-AI Interactions: A Practical Perspective From Existing Use Cases and Interviews,” in *Proceedings of the 55th Hawaii International Conference on System Sciences (HICSS 2022)*

[CR67] Hofmann P, Jöhnk J, Protschky D, Urbach N (2020) “Developing purposeful AI use cases - a structured method and its application in project management,” in *Proceedings of the 15th International Conference on Wirtschaftsinformatik (WI 2020)*, pp. 9–11

[CR68] Hofmann P, Rückel T, Urbach N (2021) “Innovating with Artificial Intelligence: Capturing the Constructive Functional Capabilities of Deep Generative Learning,” in *Proceedings of the 54th Hawaii International Conference on System Sciences (HICSS 2021)* (doi: 10.24251/HICSS.2021.669)

[CR69] Hossain M, Kauranen I (2015). Crowdsourcing: a comprehensive literature review. Strategic Outsourcing: An International Journal.

[CR70] Hosseini M, Phalp K, Taylor J, Ali R (2015) “On the Configuration of Crowdsourcing Projects,” *International Journal of Information System Modeling and Design* (6:3, SI), pp. 27–45 (doi: 10.4018/IJISMD.2015070102)

[CR71] Howe J (2006a) “Crowdsourcing: a definition,” available at https://crowdsourcing.typepad.com/cs/2006/06/crowdsourcing_a.html

[CR72] Howe J (2006). The rise of crowdsourcing. Wired magazine.

[CR73] Iansiti M, Lakhani KR (2020) Competing in the Age of AI. Harvard Business Review Press

[CR74] Introne J, Laubacher R, Olson G, Malone T (2011) “The Climate CoLab: large scale model-based collaborative planning,” in *International Conference on Collaboration Technologies and Systems*, pp. 40–47 (doi: 10.1109/CTS.2011.5928663)

[CR75] Introne J, Laubacher R, Olson G, Malone T (2013). Solving Wicked Social Problems with Socio-computational Systems. KI - Künstliche Intelligenz.

[CR76] Ito T, Hadfi R, Suzuki S (2021). An Agent that Facilitates Crowd Discussion. Group Decis Negot.

[CR77] Jalowski M, Fritzsche A, Möslein KM (2019). Facilitating collaborative design: a toolkit for integrating persuasive technologies in design activities. Procedia CIRP.

[CR78] Jespersen KR (2018). Crowdsourcing design decisions for optimal integration into the company innovation system. Decis Support Syst.

[CR79] Kamoun F, Alhadidi D, Maamar Z (2015). Weaving Risk Identification into Crowdsourcing Lifecycle. Procedia Comput Sci.

[CR80] Kampf CE (2019). Intermingling AI and IoT affordances: the expansion of social opportunities for service users and providers. Scandinavian J Inform Syst.

[CR81] Kaplan A, Haenlein M (2019). Siri, Siri, in my hand: Who’s the fairest in the land? On the interpretations, illustrations, and implications of artificial intelligence. Bus Horiz.

[CR82] Keller R, Stohr A, Fridgen G, Lockl J, Rieger A (2019) “Affordance-experimentation-actualization theory in artificial intelligence research - a predictive maintenance story,” in *Proceedings of the 40th International Conference on Information Systems (ICIS 2019)*, Association for Information Systems

[CR83] Khalifa M, Kwok R-W, Davison R (2002). The effects of process and content facilitation restrictiveness on GSS-mediated collaborative learning. Group Decis Negot.

[CR84] Kim S, Robert LP (2019) “Crowdsourcing Coordination: A Review and Research Agenda for Crowdsourcing Coordination Used for Macro-tasks,” in *Macrotask Crowdsourcing*, V.-J. Khan, K. Papangelis, I. Lykourentzou and P. Markopoulos (eds.), pp. 17–43 (doi: 10.1007/978-3-030-12334-5_2)

[CR85] Kiruthika U, Somasundaram TS, Raja SKS (2020). Lifecycle Model of a Negotiation Agent: A Survey of Automated Negotiation Techniques. Group Decis Negot.

[CR86] Kittur A, Nickerson JV, Bernstein M, Gerber E, Shaw A, Zimmerman J, Lease M, Horton J (2013) “The future of crowd work,” in *Proceedings of the 2013 conference on Computer supported cooperative work*, p. 1301 (doi: 10.1145/2441776.2441923)

[CR87] Kocsis D, Vreede G-Jde (2016) “Towards a taxonomy of ethical considerations in crowdsourcing,” in *Proceedings of the 22nd Americas Conference on Information Systems (AMCIS 2016)*, Association for Information Systems

[CR88] Kohler T, Chesbrough H (2019). From collaborative community to competitive market: the quest to build a crowdsourcing platform for social innovation. R & D Management.

[CR89] Kolfschoten GL, den Hengst-Bruggeling M, Vreede G-Jde (2007). Issues in the design of facilitated collaboration processes. Group Decis Negot.

[CR90] Kolfschoten GL, Grünbacher P, Briggs RO (2011). Modifiers for quality assurance in group facilitation. Group Decis Negot.

[CR91] Laengle S, Modak NM, Merigo JM, Zurita G (2018). Twenty-five years of group decision and negotiation: a bibliometric overview. Group Decis Negot.

[CR92] Leal Filho W, Wall T, Rui Mucova SA, Nagy GJ, Balogun A-L, Luetz JM, Ng AW, Kovaleva M, Azam S, Alves FM, Guevara F, Matandirotya Z, Skouloudis NR, Tzachor A, Malakar A, Gandhi O (2022). Deploying artificial intelligence for climate change adaptation. Technol Forecast Soc Chang.

[CR93] Lehrer C, Wieneke A, vom Brocke J, Jung R, Seidel S (2018).

[CR94] Leimeister JM (2010). Collective intelligence. Bus Inform Syst Eng.

[CR95] Leonardi (2011). When Flexible Routines Meet Flexible Technologies: Affordance, Constraint, and the Imbrication of Human and Material Agencies. MIS Q.

[CR96] Liu S, Xia F, Zhang J, Pan W, Zhang Y (2016). Exploring the trends, characteristic antecedents, and performance consequences of crowdsourcing project risks. Int J Project Manage.

[CR97] Lopez M, Vukovic M, Laredo J (2010) “Peoplecloud service for enterprise crowdsourcing,” in *IEEE International Conference on Services Computing*, pp. 538–545 (doi: 10.1109/SCC.2010.74)

[CR98] Lykourentzou I, Khan V-J, Papangelis K, Markopoulos P (2019) “Macrotask crowdsourcing: an integrated definition,” in *Macrotask Crowdsourcing*, V.-J. Khan, K. Papangelis, I. Lykourentzou and P. Markopoulos (eds.), pp. 1–13 (doi: 10.1007/978-3-030-12334-5_1)

[CR99] Maedche A, Legner C, Benlian A, Berger B, Gimpel H, Hess T, Hinz O, Morana S, Söllner M (2019). AI-based digital assistants. Bus Inform Syst Eng.

[CR100] Maister DH, Lovelock CH (1982). Managing facilitator services. Sloan Manag Rev.

[CR101] Malhotra A, Majchrzak A, Lyytinen K (2021). Socio-Technical Affordances for Large-Scale Collaborations: Introduction to a Virtual Special Issue. Organ Sci.

[CR102] Malone TW, Laubacher R, Dellarocas C (2010). The collective intelligence genome. MIT Sloan Management Review.

[CR103] Manyika J, Lund S, Bughin J, Woetzel JR, Stamenov K, Dhingra D (2016) Digital globalization: The new era of global flows. McKinsey Global Institute San Francisco

[CR104] Markus ML, Silver M (2008). A Foundation for the Study of IT Effects: A New Look at DeSanctis and Poole’s Concepts of Structural Features and Spirit. J Association Inform Syst.

[CR105] McCardle-Keurentjes M, Rouwette EAJA (2018). Asking questions: a sine qua non of facilitation in decision support?. Group Decis Negot.

[CR106] McGahan AM, Bogers MLAM, Chesbrough H, Holgersson M (2021). Tackling Societal Challenges with Open Innovation. Calif Manag Rev.

[CR107] Myers MD, Newman M (2007). The qualitative interview in IS research: examining the craft. Inf Organ.

[CR108] Nagar Y, Boer P, Garcia B (2016) A. C. “Accelerating the review of complex intellectual artifacts in crowdsourced innovation challenges,” in *Proceedings of the 37th International Conference on Information Systems (ICIS 2016)*, Association for Information Systems

[CR109] Nascimento AM, da Cunha MAlexandraV, Cortez S, de Meirelles F, Scornavacca E, de Melo VV (2018) “A literature analysis of research on artificial intelligence in management information system (MIS),” in *Proceedings of the 24th Americas Conference on Information Systems (AMCIS 2018)*, Association for Information Systems

[CR110] Nguyen C, Oh O, Kocsis D, Vreede G-J (2013) “Crowdsourcing as lego: unpacking the building blocks of crowdsourcing collaboration processes,” in *Proceedings of the 34th International Conference on Information Systems (ICIS 2013)*, Association for Information Systems

[CR111] Nguyen C, Tahmasbi N, de Vreede T, de Vreede G-J, Oh O, Reiter-Palmon R (2015) “Participant Engagement in Community Crowdsourcing,” in *Proceedings of the 23th European Conference on Information Systems (ECIS 2015)*, Association for Information Systems

[CR112] Norman DA (1999). Affordance, conventions, and design. Interactions.

[CR113] Onuchowska A, de Vreede G-J (2018) “Disruption and Deception in Crowdsourcing: Towards a Crowdsourcing Risk Framework,” in *Proceedings of the 51st Hawaii International Conference on System Sciences (HICSS 2018)* (doi: 10.24251/HICSS.2018.498)

[CR114] Ooms W, Piepenbrink R (2021). Open Innovation for Wicked Problems: Using Proximity to Overcome Barriers. Calif Manag Rev.

[CR115] Ostern N, Rosemann M (2021) “A Framework for Digital Affordances,” in *Proceedings of the 29th European Conference on Information Systems (ECIS 2021)*, Association for Information Systems

[CR116] Pedersen J, Kocsis D, Tripathi A, Tarrell A, Weerakoon A, Tahmasbi N, Xiong J, Deng W, Oh O, de Vreede G-J (2013) “Conceptual foundations of crowdsourcing: a review of IS research,” in *Proceedings of the 46th Hawaii International Conference on System Sciences (HICSS 2013)*, pp. 579–588 (doi: 10.1109/HICSS.2013.143)

[CR117] Pohlisch J (2021) “Managing the Crowd: A Literature Review of Empirical Studies on Internal Crowdsourcing,” in *Internal Crowdsourcing in Companies*, pp. 27–53 (doi: 10.1007/978-3-030-52881-2_3)

[CR118] Pumplun L, Tauchert C, Heidt M (2019) “A new organizational chassis for artificial intelligence-exploring organizational readiness factors,” in *Proceedings of the 27th European Conference on Information Systems (ECIS 2019)*, Association for Information Systems

[CR119] Qiao L, Tang F, Liu J (2018) “Feedback based high-quality task assignment in collaborative crowdsourcing,” in *IEEE 32nd International Conference on Advanced Information Networking and Applications*, pp. 1139–1146 (doi: 10.1109/AINA.2018.00163)

[CR120] Rai A (2020). Explainable AI: from black box to glass box. J Acad Mark Sci.

[CR121] Rai A, Constantinides P, Sarker S (2019) “Next Generation Digital Platforms: Toward Human-AI Hybrids,” *MIS Quarterly* (43:1), pp. iii-ix

[CR122] Retelny D, Robaszkiewicz S, To A, Lasecki WS, Patel J, Rahmati N, Doshi T, Valentine M, Bernstein MS (2014) “Expert crowdsourcing with flash teams,” in *Proceedings of the 27th annual ACM symposium on User interface software and technology*, pp. 75–85 (doi: 10.1145/2642918.2647409)

[CR123] Rhyn M, Blohm I (2017) “Combining collective and artificial intelligence: towards a design theory for decision support in crowdsourcing,” in *Proceedings of the 25th European Conference on Information Systems (ECIS 2017)*, Association for Information Systems

[CR124] Rhyn M, Blohm I, Leimeister JM (2017) “Understanding the emergence and recombination of distant knowledge on crowdsourcing platforms,” in *Proceedings of the 38th International Conference on Information Systems (ICIS 2017)*, Association for Information Systems

[CR125] Rhyn M, Leicht N, Blohm I, Leimeister JM (2020) “Opening the Black Box: How to Design Intelligent Decision Support Systems for Crowdsourcing,” in *Proceedings of the 15th International Conference on Wirtschaftsinformatik (WI 2020)*, pp. 50–65

[CR126] Riedl C, Woolley AW (2017). Teams vs. crowds: a field test of the relative contribution of incentives, member ability, and emergent collaboration to crowd-based problem solving performance. Acad Manage Discoveries.

[CR127] Rippa P, Quinto I, Lazzarotti V, Pellegrini L (2016). Role of innovation intermediaries in open innovation practices: differences between micro-small and medium-large firms. Int J Bus Innov Res.

[CR128] Robert LP (2019) “Crowdsourcing controls: a review and research agenda for crowdsourcing controls used for macro-tasks,” in *Macrotask Crowdsourcing*, V.-J. Khan, K. Papangelis, I. Lykourentzou and P. Markopoulos (eds.), pp. 45–126 (doi: 10.1007/978-3-030-12334-5_3)

[CR129] Russell SJ, Norvig P (2021) *Artificial intelligence*: *A modern approach*, Hoboken: Pearson

[CR130] Rzepka C, Berger B (2018) “User interaction with AI-enabled systems: a systematic review of IS research,” in *Proceedings of the 39th International Conference on Information Systems (ICIS 2018)*, Association for Information Systems

[CR131] Schenk E, Guittard C (2011). Towards a characterization of crowdsourcing practices. J Innov Econ.

[CR132] Schlagwein D, Cecez-Kecmanovic D, Hanckel B (2019). Ethical norms and issues in crowdsourcing practices: a Habermasian analysis. Inform Syst J.

[CR133] Schmitz H, Lykourentzou I (2018). Online sequencing of non-decomposable macrotasks in expert crowdsourcing. ACM Trans Social Comput.

[CR134] Schoormann T, Strobel G, Möller F, Petrik D (2021) “Achieving Sustainability with Artificial Intelligence - A Survey of Information Systems Research,” in *Proceedings of the 42nd International Conference on Information Systems (ICIS 2021)*, Association for Information Systems

[CR135] Schreier M (2012) Qualitative content analysis in practice. Sage publications

[CR136] Schultze U, Avital M (2011). Designing interviews to generate rich data for information systems research. Inf Organ.

[CR137] Seeber I, Bittner E, Briggs RO, de Vreede G-J, de Vreede T, Druckenmiller D, Maier R, Merz AB, Oeste-Reiß S, Randrup N (2018) and others. “Machines as teammates: A collaboration research agenda,” in *Proceedings of the 51st Hawaii International Conference on System Sciences (HICSS 2018)*

[CR138] Seeber I, Bittner E, Briggs RO, de Vreede T, de Vreede G-J, Elkins A, Maier R, Merz AB, Oeste-Reiß S, Randrup N, Schwabe G, Söllner M (2020) Machines as teammates: a research agenda on AI in team collaboration. 57:103174. 10.1016/j.im.2019.103174). 2

[CR139] Seeber I, Waizenegger L, Demetz L, Merz AB, de Vreede G-J, Maier R, Weber B (2016) “IT-supported formal control: how perceptual (in) congruence affects the convergence of crowd-sourced ideas,” in *Proceedings of the 37th International Conference on Information Systems (ICIS 2016)*, Association for Information Systems

[CR140] Shafiei Gol E, Stein M-K, Avital M (2019). Crowdwork platform governance toward organizational value creation. J Strateg Inf Syst.

[CR141] Siemon D (2022). Elaborating Team Roles for Artificial Intelligence-based Teammates in Human-AI Collaboration. Group Decis Negot.

[CR142] Simon HA (1995). Artificial intelligence: an empirical science. Artif Intell.

[CR143] Sonnenberg C, vom Brocke J, Helfert M, Donnellan B (2012). “Evaluation patterns for design science research artefacts. Practical Aspects of Design Science.

[CR144] Sousa MJ, Rocha Á (2020). Decision-Making and Negotiation in Innovation & Research in Information Science. Group Decis Negot.

[CR145] Steffen JH, Gaskin JE, Meservy TO, Jenkins JL, Wolman I (2019). Framework of Affordances for Virtual Reality and Augmented Reality. J Manage Inform Syst.

[CR146] Stone P, Brooks R, Brynjolfsson E, Calo R, Etzioni O, Hager G, Hirschberg J, Kalyanakrishnan S, Kamar E, Kraus S (2016) and others. “Artificial intelligence and life in 2030,” *One Hundred Year Study on Artificial Intelligence: Report of the 2015–2016 Study Panel*, p. 52

[CR147] Suthers DD (2006). Technology affordances for intersubjective meaning making: A research agenda for CSCL. Int J Computer-Supported Collaborative Learn.

[CR148] Tavanapour N, Bittner EAC (2018a) “Automated facilitation for idea platforms: design and evaluation of a chatbot prototype,” in *Proceedings of the 39th International Conference on Information Systems (ICIS 2018)*, Association for Information Systems

[CR149] Tavanapour N, Bittner EAC (2018b) “The Collaboration of Crowd Workers,” *Research-in-Progress Papers*

[CR150] Tazzini G, Montelisciani G, Gabelloni D, Paganucci S, Fantoni G (2013) “A structured team building method for collaborative crowdsourcing,” in *2013 International Conference on Engineering, Technology and* Innovation *(ICE) & IEEE International Technology Management Conference*, IEEE, pp. 1–11 (doi: 10.1109/ITMC.2013.7352708)

[CR151] Te’eni D, Avital M, Hevner A, Schoop M, Schwartz D (2019) “It Takes Two to Tango: Choreographing the Interactions between Human and Artificial Intelligence,” in *Proceedings of the 27th European Conference on Information Systems (ECIS 2019)*, Association for Information Systems

[CR152] Toubia O, Netzer O (2017). Idea Generation, Creativity, and Prototypicality. Mark Sci.

[CR153] Troll J, Naef S, Blohm I (2017) *A Mixed Method Approach to Understanding Crowdsourcees’ Engagement Behavior*, available at https://aisel.aisnet.org/icis2017/HumanBehavior/Presentations/34

[CR154] United Nations (2015) “Transforming our world: the 2030 agenda for sustainable development,&#8221

[CR155] Valentine MA, Retelny D, To A, Rahmati N, Doshi T, Bernstein MS(2017) “Flash Organizations: Crowdsourcing Complex Work by Structuring Crowds As Organizations,” in *Proceedings of the 2017 CHI Conference on Human Factors in Computing Systems*, pp. 3523–3537 (doi: 10.1145/3025453.3025811)

[CR156] Vianna F, Peinado J, Graeml AR(2019) “Crowdsourcing platforms: objective, activities and motivation,” in *Proceedings of the 25th Americas Conference on Information Systems (AMCIS 2019)*, Association for Information Systems

[CR157] Vivacqua AS, Marques LC, Ferreira MS, de Souza JM (2011). Computational indicators to assist meeting facilitation. Group Decis Negot.

[CR158] Volkoff O, Strong DM (2013). Critical realism and affordances: theorizing IT-associated organizational change processes. MIS Q.

[CR159] Volkoff O, Strong DM(2017) “Affordance theory and how to use it in IS research,”The Routledge companion to management information systems, pp.232–245

[CR160] vom Brocke J, Simons A, Riemer K, Niehaves B, Plattfaut R, Cleven A(2015) “Standing on the shoulders of giants: challenges and recommendations of literature search in information systems research,” *Communications of the Association for Information Systems* (37) (doi: 10.17705/1CAIS.03709)

[CR161] Vukicevic A, Vukicevic M, Radovanovic S, Delibasic B (2022) BargCrEx: A System for Bargaining Based Aggregation of Crowd and Expert Opinions in Crowdsourcing. Group Decis Negot 1–30. doi: 10.1007/s10726-022-09783-0) 10.1007/s10726-022-09783-0 PMC9123878 35615756

[CR162] Vukovic M, Bartolini C(2010) “Towards a research agenda for enterprise crowdsourcing,” in *Leveraging Applications of Formal Methods, Verification, and Validation*, T. Margaria and B. Steffen (eds.), pp. 425–434 (doi: 10.1007/978-3-642-16558-0_36)

[CR163] Vukovic M, Laredo J, Rajagopal S(2010) “Challenges and Experiences in Deploying Enterprise Crowdsourcing Service,” in *Web Engineering*, B. Benatallah, F. Casati, G. Kappel and G. Rossi (eds.)

[CR164] Vyas D, Chisalita CM, van der Veer GC(2006) “Affordance in interaction,” in *Proceedings of the 13th Eurpoean conference on Cognitive ergonomics trust and control in complex socio-technical systems*, p. 92 (doi: 10.1145/1274892.1274907)

[CR165] Wang A, Pruksachatkun Y, Nangia N, Singh A, Michael J, Hill F, Levy O, Bowman S(2019) “Superglue: A stickier benchmark for general-purpose language understanding systems,” *Advances in neural information processing systems* (32)

[CR166] Wedel M, Ulbrich H(2021) “Systematization Approach for the Development and Description of an Internal Crowdsourcing System,” in *Internal Crowdsourcing in Companies*, pp. 55–78 (doi: 10.1007/978-3-030-52881-2_4)

[CR167] Wiggins A, Crowston K(2011) “From Conservation to Crowdsourcing: A Typology of Citizen Science,” in *2011 44th Hawaii International Conference on System Sciences*, Kauai, HI. 04.01.2011–07.01.2011, IEEE, pp. 1–10 (doi: 10.1109/HICSS.2011.207)

[CR168] Wilson HJ, Daugherty PR (2018). Collaborative intelligence: humans and AI are joining forces. Harvard Business Rev.

[CR169] Winkler R, Briggs RO, de Vreede G-J, Leimeister JM, Oeste-Reiss S, Sollner M (2020) Modeling Support for Mass Collaboration in Open Innovation Initiatives—The Facilitation Process Model 2.0. IEEE Trans Eng Manage 1–15. doi: 10.1109/TEM.2020.2975938)

[CR170] Wolfswinkel JF, Furtmueller E, Wilderom CPM (2013). Using grounded theory as a method for rigorously reviewing literature. Eur J Inform Syst.

[CR171] Xia F, Liu S, Zhang J(2015) “How Social Subsystem and Technical Subsystem Risks Influence Crowdsourcing Performance,” in *Proceedings of the 19th Pacific Asia Conference on Information Systems (PACIS 2015)*, Association for Information Systems

[CR172] Xiang W, Sun L, You W, Yang C (2018). Crowdsourcing intelligent design. Front Inform Technol Electron Eng.

[CR173] Yin RK (2018). *Case study research and applications*: *design and methods*.

[CR174] Zajonc RB (1965). Social facilitation. Sci (New York N Y).

[CR175] Zhao Y, Zhu Q (2014). Evaluation on crowdsourcing research: current status and future direction. Inform Syst Front.

[CR176] Zhao Y, Zhu Q (2016). Conceptualizing task affordance in online crowdsourcing context. Online Inf Rev.

[CR177] Zheng Q, Wang W, Yu Y, Pan M, Shi X(2017) “Crowdsourcing complex task automatically by workflow technology,” in *Management of Information, Process and Cooperation*, J. Cao and J. Liu (eds.), pp. 17–30 (doi: 10.1007/978-981-10-3996-6_2)

[CR178] Zogaj S, Bretschneider U(2014) “Analyzing governance mechanisms for crowdsourcing information systems: a multiple case analysis,” in *Proceedings of the 22nd European Conference on Information Systems (ECIS 2014)*, Association for Information Systems

[CR179] Zogaj S, Leicht N, Blohm I, Bretschneider U(2015) “Towards Successful Crowdsourcing Projects: Evaluating the Implementation of Governance Mechanisms,” in *Proceedings of the 36th International Conference on Information Systems (ICIS 2015)*, Association for Information Systems

[CR180] Zuchowski O, Posegga O, Schlagwein D, Fischbach K (2016). Internal crowdsourcing: conceptual framework, structured review, and research agenda. J Inform Technol.

